# Multiscale causal networks identify VGF as a key regulator of Alzheimer’s disease

**DOI:** 10.1038/s41467-020-17405-z

**Published:** 2020-08-07

**Authors:** Noam D. Beckmann, Wei-Jye Lin, Minghui Wang, Ariella T. Cohain, Alexander W. Charney, Pei Wang, Weiping Ma, Ying-Chih Wang, Cheng Jiang, Mickael Audrain, Phillip H. Comella, Amanda K. Fakira, Siddharth P. Hariharan, Gillian M. Belbin, Kiran Girdhar, Allan I. Levey, Nicholas T. Seyfried, Eric B. Dammer, Duc Duong, James J. Lah, Jean-Vianney Haure-Mirande, Ben Shackleton, Tomas Fanutza, Robert Blitzer, Eimear Kenny, Jun Zhu, Vahram Haroutunian, Pavel Katsel, Sam Gandy, Zhidong Tu, Michelle E. Ehrlich, Bin Zhang, Stephen R. Salton, Eric E. Schadt

**Affiliations:** 1 Department of Genetics and Genomic Sciences, Icahn School of Medicine at Mount Sinai, One Gustave L. Levy Place, New York, NY 10029 USA; 2 Icahn Institute of Genomics and Multiscale Biology, Icahn School of Medicine at Mount Sinai, One Gustave L. Levy Place, New York, NY 10029 USA; 3 Graduate School of Biomedical Sciences, Icahn School of Medicine at Mount Sinai, New York, NY USA; 4 Guangdong Provincial Key Laboratory of Malignant Tumor Epigenetics and Gene Regulation, Medical Research Center, Sun Yat-Sen Memorial Hospital, Sun Yat-Sen University, 510120 Guangzhou, China; 5 Guangdong Province Key Laboratory of Brain Function and Disease, Zhongshan School of Medicine, Sun Yat-sen University, Guangzhou, Guangdong 510080 China; 6 Nash Family Department of Neuroscience, Icahn School of Medicine at Mount Sinai, One Gustave L. Levy Place, New York, NY 10029 USA; 7 Department of Neuroscience, Friedman Brain Institute, Icahn School of Medicine at Mount Sinai, One Gustave L. Levy Place, New York, NY 10029 USA; 8 Department of Neurosurgery, Icahn School of Medicine at Mount Sinai, One Gustave L. Levy Place, New York, NY 10029 USA; 9 Center for Statistical Genetics, Icahn School of Medicine at Mount Sinai, New York, NY USA; 10 Charles Bronfman Institute of Personalized Medicine, Icahn School of Medicine at Mount Sinai, New York, NY USA; 11 Department of Neurology and Center for Neurodegenerative Disease, Emory University School of Medicine, Atlanta, GA USA; 12 Department of Biochemistry, Emory University School of Medicine, Atlanta, GA USA; 13 Department of Neurology, Icahn School of Medicine at Mount Sinai, One Gustave L. Levy Place, New York, NY 10029 USA; 14 Alzheimer’s Disease Research Center, Icahn School of Medicine at Mount Sinai, New York, NY 10029 USA; 15 Department of Pharmacological Sciences, Icahn School of Medicine at Mount Sinai, One Gustave L. Levy Place, New York, NY 10029 USA; 16 Department of Psychiatry, Icahn School of Medicine at Mount Sinai, One Gustave L. Levy Place, New York, NY 10029 USA; 17 Sema4, Stamford, CT 06902 USA; 18 Department of Psychiatry, JJ Peters VA Medical Center, 130 West Kingsbridge Road, Bronx, NY 10468 USA; 19 Department of Pediatrics, Icahn School of Medicine at Mount Sinai, One Gustave L. Levy Place, New York, NY 10029 USA

**Keywords:** Biological techniques, Bioinformatics, Gene expression analysis, Mass spectrometry, Proteomic analysis

## Abstract

Though discovered over 100 years ago, the molecular foundation of sporadic Alzheimer’s disease (AD) remains elusive. To better characterize the complex nature of AD, we constructed multiscale causal networks on a large human AD multi-omics dataset, integrating clinical features of AD, DNA variation, and gene- and protein-expression. These probabilistic causal models enabled detection, prioritization and replication of high-confidence master regulators of AD-associated networks, including the top predicted regulator, VGF. Overexpression of neuropeptide precursor VGF in 5xFAD mice partially rescued beta-amyloid-mediated memory impairment and neuropathology. Molecular validation of network predictions downstream of VGF was also achieved in this AD model, with significant enrichment for homologous genes identified as differentially expressed in 5xFAD brains overexpressing VGF. Our findings support a causal role for VGF in protecting against AD pathogenesis and progression.

## Abstract (web summary)

To investigate the molecular foundation of sporadic Alzheimer’s disease (AD), Beckmann et al. constructed multiscale causal networks on a large human AD multi-omics dataset, detecting AD-associated networks and their top predicted regulator, VGF, with extensive validation in the 5xFAD mouse model.

## Introduction

Late-onset Alzheimer’s disease (AD) results in progressive loss of cognitive function and memory, affects more than 5.8 million Americans, and its incidence is projected to double in the next 20 years^1^. The brains of AD patients have hallmark senile plaques in the neuropil and around brain blood vessels, composed of accumulated amyloid beta (Aβ) and neurofibrillary tangles (NFT) inside neurons, which comprises microtubule-associated hyperphosphorylated Tau protein^2^. While therapeutic strategies targeting Aβ and Tau pathologies have been aggressively pursued, failure to deliver efficacious treatments has increased the urgency to identify different mechanisms underlying AD, including a focus on the immune system, through microglial cells, that has been shown to play a key role in AD^3–9^.

Genome-wide association studies (GWAS) have identified over 20 AD risk loci falling mainly in noncoding regions of the genome^10–13^, revealing a complex neurobiology with no single genetic cause. For most AD risk loci, target gene(s) and their pathways are difficult to identify and validate, and the broader networks they form remain largely uncharacterized. Integrative biology approaches, combining large-scale, high-dimensional data (e.g., DNA variation, and gene and protein expression) generated in disease and control cohorts, complement GWAS-like approaches by employing advanced computational modeling techniques that incorporate multiple levels of data into probabilistic causal models of disease (or wellness). These enable molecular traits correlated with disease to be distinguished from those that are causally related (Supplementary Table 1). These causal relationships can be inferred with enhanced power by incorporating DNA-based variations (expression quantitative trait loci, eQTL) as a systematic perturbation source (Supplementary Table 1). By integrating DNA variation with additional types of molecular and clinical data, more complex, holistic models of disease can be constructed and mined to elucidate regulatory and mechanistic drivers of disease and points of therapeutic intervention.

Here, we employed probabilistic causal reasoning to organize different scales of data (DNA, RNA, protein, and clinical data) generated as part of the Accelerating Medicines Partnership-AD (AMP-AD; https://www.synapse.org/#!Synapse:syn2580853/wiki/409840) on a population of late-onset AD individuals and controls, to construct a predictive “multiscale” network model of AD that provides a comprehensive characterization of the complex architecture of AD in the human brain. Causal links among nodes comprising these multiscale networks can be mined to identify gene- or protein-expression traits whose changes in expression are predicted to modulate network states driving AD. Identification of these causal regulators of disease networks provides an objective, data-driven way to uncover novel key drivers (KDs) of disease. Strikingly, among the KDs we identified was *VGF*, a nerve growth factor (NGF) and a brain-derived neurotrophic factor (BDNF)-inducible gene encoding a protein and neuropeptide precursor, the actions of which are in part BDNF/TrkB dependent^14,15^. Although VGF has been reported to regulate fear and spatial memories in mouse models^14,16,17^, and has previously been shown to correlate with AD (VGF-derived peptides are reduced in cerebral spinal fluid (CSF) of AD patients)^18–22^, VGF has not previously been causally associated with AD. We determined through our network models that VGF was the only downregulated KD for AD that was conserved across the RNA, protein, and combined RNA and protein networks we constructed. We replicated these findings in other brain regions^23^ and in an independent dataset^24,25^, and observed association of *VGF* expression to the genome-wide risk for AD in the I-GAP (The International Genomics of Alzheimer’s Project) AD GWAS^10^. Utilizing three independent models of VGF overexpression in the 5xFAD mouse model of familial AD (FAD), we provide molecular and functional validation of our multiscale causal network analyses, and conclude that *VGF* is a KD of AD pathophysiology, and that *VGF*-linked genes and clinical features provide novel insights into the mechanisms underlying AD risk and pathogenesis.

## Results

### Overview of strategy

**Fig. 1:**
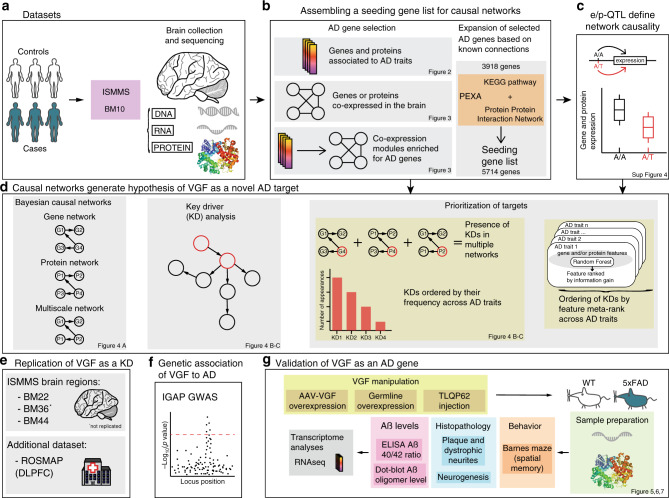
Pipeline overview. Large-scale, high-dimensional datasets generated in hundreds of subjects serve as the input into our integrative pipeline (**a**), which comprises a series of steps that first generate the appropriate input features for causal network reconstructions (**b**, **c**), then network reconstruction and identification of key driver genes (**d**), and finally validation via three independent paths: replication (**e**), human genetic association (**f**), and experimental disease model (**g**).

Our overall strategy for elucidating the complexity of AD is depicted in Fig. 1 (and in Supplementary Fig. 1) and is centered on the objective, data-driven construction of causal network models of AD that can be queried to identify components associated with AD. The causal regulators modulating the state of these AD-associated network components can be readily identified from the network model. We previously developed and applied network reconstruction algorithm, RIMBANET, which statistically infers causal relationships between DNA variation, gene expression, protein expression, and clinical features scored in hundreds of individuals (Supplementary Table 1). The inputs required for these analyses are molecular (i.e., genotype and gene or protein expression) and clinical data, and direct relationships between them, such as QTLs and causal relationships among traits inferred by causal mediation analysis that uses mapped QTL as a source of perturbation. These relationships are input to the network construction algorithm as constraints on the network topology (referred to as structure priors), boosting the power to infer causal relationships at the network level (Supplementary Table 1).

### The Mount Sinai Brain Bank (MSBB) Study population and data quality control

AD and control populations profiled in this study are part of the MSBB^23^. From the >2000 participants within MSBB, 143 definitive AD cases were selected, along with 135 possible and probable AD cases, and 86 non-demented controls^23^ (Supplementary Data 1). The selection criteria were neuropathological evidence of AD by CERAD (Consortium to Establish a Registry for Alzheimer’s Disease)^26^ classification or no neuropathological evidence of AD. Donors with neuropsychiatric disease and/or comorbid neurodegenerative diseases, and/or neuropathologically significant cerebrovascular disease, were excluded. DNA, RNA, and protein were isolated from BM10 for molecular profiling (Fig. 1b). The DNA- and RNA-sequencing (RNA-seq) data were processed using standard pipelines, including quantification of gene expression, variant detection, and quality control (QC) for the RNA-seq data^27^ (“Methods”).

### Identifying an AD-centered gene set to construct a causal model of AD

**Fig. 2:**
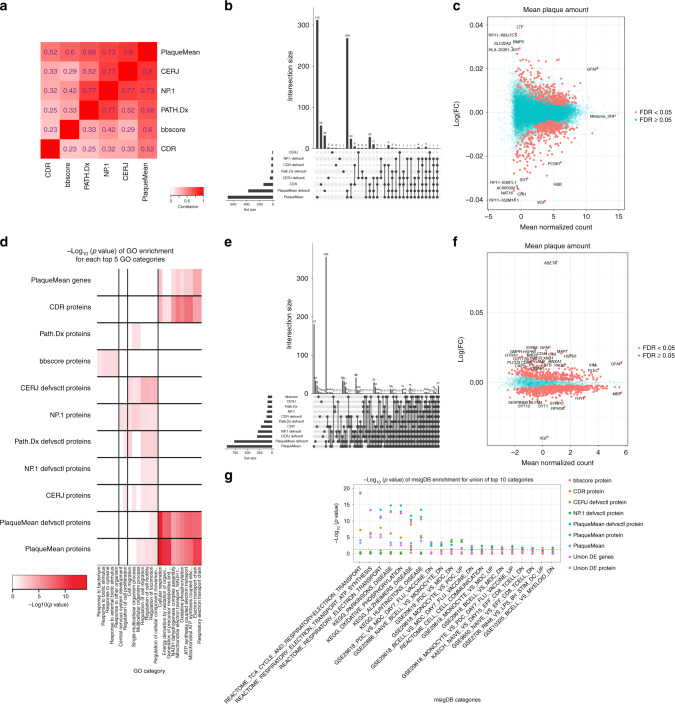
Characterization of AD traits, and brain gene and protein expression. **a** Canonical correlation heatmap of disease traits. The intensity of the red color indicates the strength of correlation between traits; the canonical correlation is indicated in each box. The *x*- and *y*-axis represent the traits: clinical dementia rating (CDR), Braak score (bbscore), clinical neuropathology (PATH.Dx), neuropathology category (NP.1), CERAD neuropath criteria (CERJ), and mean neocortical plaque density (number of plaques/mm^2^, PlaqueMean). **b**, **e** Breakdown of DE genes (**b**) and proteins (**e**). This figure shows the UpsetR plot (“Methods”) of the DE genes or proteins overlapping across tests. The bars represent the size of the DE sets and the points represent the category to which each set belongs. **c**, **f** PlaqueMean DE genes (**c**) and proteins (**f**). The *x*-axis is the mean normalized count for each gene or protein and the *y*-axis is their log fold change. Blue and red genes and proteins correspond to FDR ≥ 0.05 and FDR < 0.05, respectively. The strongest DE genes are highlighted on the plot. **d** GO term enrichment across all signatures. The heatmap depicted represents the −log 10(FDR) of the top 5 most significant GO terms associated with signatures across all traits. Rows are GO terms and columns signatures. **g** MsigDB pathway enrichment for all signatures. The barplot represents the union of the top 10 significant MsigDB categories associated to signatures for all traits; *x*- and *y*-axis are MsigDB terms and the −log 10(FDR), respectively. Colors represent the individual traits.

To construct AD-centered causal network models, we constrained the number of inputs into the reconstruction process to those supported by the MSBB data as associating with AD. This reduction in dimensionality also provided a computationally tractable path for the network constructions. We first identified gene- and protein-expression traits associated with AD (Fig. 1b). To cast the most comprehensive net for AD-associated features, we examined the association between molecular-expression traits and clinical/neuropathological features used to characterize AD. Given the complexity of AD, six clinical and neuropathologic characteristics were used to define the severity of disease in patients, including clinical staging with clinical dementia rating (CDR), pathological staging of NFTs or Braak score (bbscore), clinical neuropathology diagnosis (PATH.Dx), CERAD neuropath criteria (CERJ), neuropathology category (NP-1), and mean cortical neuritic plaque density (PlaqueMean). We characterized differences and similarities specific to each of these disease traits by examining their canonical correlation structure with one another in the MSBB population (Fig. 2a). While they were highly correlated, visible variation among them highlights their complementary nature, with nonoverlapping signals that may represent different aspects or subtypes of AD (Fig. 2a). Thus, we constructed differential expression (DE) signatures for each of these clinical AD features.

As defined in Supplementary Table 2, we computed DE signatures for AD by comparing controls against individuals with any level of dementia or pathology, and then controls against individuals with neuropathologically proven AD (definite AD). This way, we generated signatures across the range of disease. We detected significant DE signatures at a false discovery rate (FDR) < 0.05 for most traits across the disease spectrum (Figs. 1b and 2b). The PlaqueMean disease trait generated the largest DE signature (Fig. 2c, d), with Gene Ontology (GO) term “respiratory electron transport chain” (fold enrichment (FE) = 4.9, FDR = 4.42e − 5) as the most enriched pathway. From the log fold-change (log(FC)) distribution (Fig. 2c) of PlaqueMean signature, the gene *VGF*, NGF inducible, has the largest negative log(FC) (more highly expressed in controls than cases). VGF was previously shown to be downregulated in patients with FAD^28^, which is consistent with our findings here. DE signatures for other disease traits (Supplementary Data 2) are depicted in Supplementary Fig. 2a.

We also ran DE analysis to identify AD signatures from the protein-expression data (Fig. 2e, Fig. 1b, Supplementary Fig. 2b) and found that significant associations were identified for all AD clinical features, with PlaqueMean giving rise to the most significant signature (Fig. 2e). For each clinical and neuropathological trait, the protein with the highest log(FC) was Aβ (mass spectrometry measurement, referred throughout the paper as Aβ), followed by other known AD proteins such as MAPT, GFAP, HSPB1, RPH3A, SYT1, and PADI2 (Supplementary Table 3). Strikingly, as with the gene DE signature, the protein with the lowest log(FC) was VGF, highlighting the strong dysregulation of the gene/protein product in AD brains (Fig. 2f). Several protein DE signatures were enriched for GO terms (Fig. 2d), with “cellular respiration” as the most significant one for the PlaqueMean protein DE signature (FDR = 8.3e − 15, FE = 2.4). Electron transport chain and AD KEGG (Kyoto Encyclopedia of Genes and Genomes) pathways from MsigDB (The Molecular Signatures Database) and KEGG databases, respectively, were also significantly enriched for the PlaqueMean protein DE signature (Fig. 2g, Supplementary Data 2).

To validate the AD signal contained within our data, we compared our DE results to previously published AD gene- and protein-expression signatures, assembling published study-specific sets of DE for significantly up- and downregulated genes and proteins (Supplementary Table 1). Supplementary Figure 2c (genes) and 2d (proteins) shows the FEs of our DE products for published AD signatures, readily recapitulating them and their directionality. Additionally, despite the low number of loci associated with AD in the latest AD GWAS^13^, DE signals for a subset of our AD traits for both genes and proteins are enriched for GWAS mapped genes and for all genes in topologically associated domains that contain the genome-wide significant loci (Supplementary Fig. 2c, d), thereby confirming the validity of our signal.

The union of genes and of proteins across all DE signatures (788 genes and 1016 proteins at FDR < 0.05, respectively) formed preliminary sets of AD-associated input features for network reconstructions. Only 55 features overlapped between these two signatures, demonstrating the highly complementary nature of gene- and protein-expression data. These resulting sets of 788 genes and 1016 proteins are referred to as the AD DE signature sets.

DE analyses provide the most straightforward way to uncover patterns of expression associating with AD; however, power is limited with respect to small to moderate expression differences. To complement DE analysis to identify AD-associated genes, we clustered genes and proteins into data-driven, functional biological groups by constructing gene and protein co-expression networks (GCN and PCN), which have enhanced power to detect co-regulated sets of genes (modules) likely to be involved in common biological processes. Co-expression modules enriched for genes associated with AD implicate all genes in said module as potentially AD associated, even if they were not identified as DE.

**Fig. 3:**
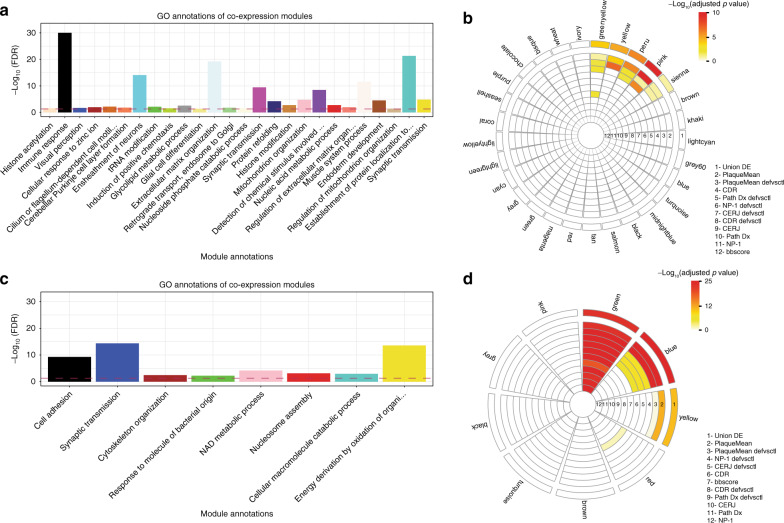
Co-expression network analyses. **a**, **c** Top GO annotations for gene (**a**) and protein (**c**) co-expression modules. The *x*- and *y*-axis represent the best GO term associated with each module and the −log 10(FDR) of the enrichment, respectively. The color of the bars represents the module names. In bold are the four modules enriched for genes in the union of the DE signatures. **b**, **d** Module enrichments for DE genes (**b**) and proteins (**d**). The circos plot depicts the enrichment of each module for DE genes or proteins; the “hotness” of the color represents the magnitude of the −log 10(adjusted *p* value) of the enrichment for the corresponding signature list. The traits are defined as 1 through 12; in bold are the modules enriched for the union of the DE signatures.

The GCN was comprised of 24,865 genes and 29 modules (Fig. 1b, Supplementary Data 3), while the PCN consisted of 2692 proteins organized into nine modules (Fig. 1b, Supplementary Data 3), with most modules (26 and 8, respectively) having significant GO term enrichments at a Bonferroni-adjusted *p* value <0.05 (Fig. 3a, c). To assess which sets of modules were associated with AD, we projected the DE signature sets onto the G/PCN modules (Figs. 1b and 3b, d). We identified four modules from the GCN with significant enrichment for the gene AD DE signature set (Fig. 3b) and for GO terms “induction of positive chemotaxis” (greenyellow, FDR = 3.0e − 2), “histone modification” (peru, FDR = 1.7e − 3), “mitochondrion organization” (pink, FDR = 1.9e − 5), and “synaptic transmission” (yellow, FDR = 1.6e − 5) (Supplementary Data 3). For the PCN, we identified three modules enriched for the protein AD DE signature set (Fig. 3d) and for “synaptic transmission” (blue, FDR = 4.6e − 15), “response to molecule of bacterial origin” (green, FDR = 5.9e − 3), and “energy derivation by oxidation of organic compounds” (yellow, FDR = 2.8e − 14) (Supplementary Data 3). We note that co-expression networks constructed by combining gene and protein expression did not result in clear connections between these data types (Supplementary information).

To form the most comprehensive set of AD-associated genes supported by our data, we expanded the DE signature set of input genes for the predictive network constructions by taking its union across all gene co-expression modules enriched for the DE signature sets, resulting in 3918 genes, referred to here as the expanded DE signature set (Fig. 1b).

### Genetic modulation of gene and protein expression in the prefrontal cortex

Integration of QTLs as a systematic source of perturbation to enhance causal inference among molecular traits, an approach we and others have demonstrated across a broad range of diseases and data types (Supplementary Table 1), is central to our approach to construct predictive network models. QTL mapping identifies DNA loci associating with quantitative traits (i.e., gene- and protein-expression), enabling the identification of regulatory and mechanistic relationships among genes and proteins, and providing critical insights into biological processes related to the functioning of cells and their association to disease. Since gene- and protein-expression traits were scored in this study, we mapped eQTL (*N* = 188 samples) and protein quantitative trait loci (pQTL) (*N* = 192 samples) for all molecular traits to identify significant QTL as additional inputs into the network reconstruction process.

We found 4224 genes with at least one eQTL (eGenes) and 158 proteins with at least one pQTL (eProteins), at FDR < 0.05 (Supplementary Data 4). To assess their degree of conservation across RNA and protein domains and to help illuminate AD genetics, we characterized the number of QTLs overlapping the expanded AD DE signatures (Fig. 1c, Supplementary Fig. 3a). Of these, we identified 83 proteins with pQTLs and 683 genes with eQTLs, including seven genes and proteins with both eQTL and pQTL. Supplementary Figure 3b exemplifies *GSTM3*, whose cortical gene- and protein-expression levels are associated with a shared single-nucleotide polymorphism (SNP), rs1332018^29^. Given the relationship between transcripts and the proteins they encode, we applied a causal mediation test^30,31^ to assess whether changes in gene expression induced by eQTLs were causal for the corresponding changes in protein expression for the 33 product pairs under control of the same SNPs. Interestingly, causal mediation supported 26 products of gene and protein expressions as being independently regulated by *cis* variation (Supplementary Data 4), suggesting that translational events may be influenced by the same *cis* variation impacting transcription, albeit in an independent fashion, perhaps partially explaining the low correlation we and others have observed between gene and protein expressions (Supplementary information).

Of the e/pQTLs identified, 766 corresponded to genes and proteins overlapping the expanded AD DE signature and were included as inputs into the network constructions.

### Identification and prioritization of KD genes identified from predictive network models of AD

To elucidate the structure of the complex interactions represented in the expanded AD DE signature set and associated QTL, we employed a Bayesian network (BN) modeling approach (Fig. 1d). BNs are graphical models that capture relationships (depicted as edges) among nodes (gene- or protein-expression traits) systematically across high-dimensional datasets. BNs not only capture linear correlations and higher-order correlations among nodes (like co-expression networks), but can also capture nonlinear relationships and infer causal links that define information flow, thereby providing a richer, more informative context for discovery (Supplementary Table 1). Because the number of possible networks to search through to identify the best data fit grows exponentially with the number of nodes included, a brute force search of all networks is not feasible^32^. Heuristics are used to constrain the size of the search space and to efficiently search it^33^. Thus, we constructed an AD-focused seeding gene set to reduce search space, with the core of this set comprised of the AD DE signatures (Supplementary Data 1) expanded to include all genes in the co-expression network modules significantly enriched for these core AD signature genes (Supplementary Data 3).

**Fig. 4:**
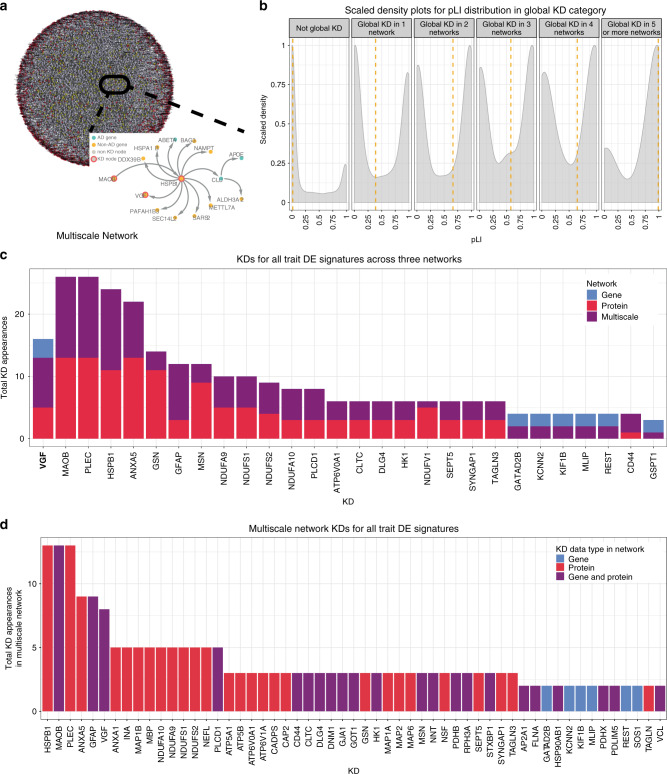
Bayesian causal networks and key drivers. **a** Full Bayesian network and an APOE subnetwork. Background visualization of the multiscale AD network described in the main text using an edge-weighted spring embedded layout. The red nodes are proteins and the blue nodes genes. Key driver (KD) genes and proteins are highlighted in yellow. Foreground multiscale subnetwork comprised of genes within a path length of 3 to APOE. Node names and properties are defined in the panel legend. **b** Density plots of the distribution of pLI scores for genes and proteins by the number of times they appear as global KDs across the three discovery and four replication networks. The yellow dashed line represents the median pLI score for that category. **c** Distribution of the number of times genes and proteins across all three discovery networks were identified as KDs across all DE signatures. The *x*- and *y*-axis depict the different KDs appearing in at least two networks and the number of times they are identified as KDs for DE signatures across all three networks. The colors of the bars are indicative of the network of origin of the KDs. **d** KD of DE signatures in the multiscale network, as described for (**b**). The color of the bars is indicative of KDs presence only in the gene expression, protein expression, or in both.

A limitation of this empirically determined gene set is that it may miss important genes due to nonlinear interactions not captured by co-expression networks, a lack of power to detect all relevant genes in the gene expression data, or genes active in tissues or stages of disease that were not as well captured in the MSBB population. To account for this, we further expanded the seeding gene set (3918 genes) with previously published knowledge using the PEXA algorithm^34^, which enables inclusion of genes from literature-derived pathways linked to the core genes or genes interacting with coding products of the core gene set in protein–protein interaction (PPI) networks. Application of PEXA resulted in the identification of 1796 additional genes, bringing our final list of genes for BN reconstructions to 5714 genes (Fig. 1b, Supplementary Data 3), compared to 24,865 transcripts expressed in the dataset. This final gene list included previously identified AD GWAS genes (six genes and five protein products)^10^, thereby integrating AD genetics into our causal network models that further implicate causal genes for AD. From the seeding gene set we constructed three BNs, one for each data type and one multiscale BN combining gene and protein expressions (Figs. 1d and  4a, Supplementary Data 3). Figure 4a illustrates subnetwork structure around AD risk factor apolipoprotein E (APOE), in addition to other AD genes.

Since the resulting BNs infer the causal flow of information, they can be queried to find major points of regulatory control (Supplementary Table 1). We analyzed each BN to catalog master causal regulators (referred to as key driver genes/proteins, or KDs) predicted by the network to modulate its state. Key driver analysis (KDA)^35^ was developed to identify network nodes predicted to either modulate a significantly enriched proportion of nodes comprising a subnetwork of interest (local KD), or a larger number of downstream targets outside the local network neighborhood (global KD). To ascertain the global structure of the networks with respect to their predicted global KD, we assessed whether network structures were validated by known biology. We consider a KD as molecularly validated if network genes predicted to change in response to changes in the KD significantly overlap genes observed to change in an experimental perturbation carried out on the KD. For this, we used published and curated single-gene perturbation experiments from the Enrichr database^36^, extracting 341 unique single-gene perturbations from 420 gene expression signatures in tissues relevant to AD (central nervous system (CNS) and immune system). While these perturbations were performed in several models and under different conditions, we found that up to 40% of KDs had predicted gene perturbation signatures that were significantly overlapping (FDR < 0.05) with the corresponding perturbation signatures (Supplementary Fig. 3c, e), validating the predictive power of our network. Interestingly, protein network KDs were not validated by gene expression signatures represented in the Enrichr database, potentially reflecting the differences described earlier between gene and protein expressions. As previously shown^37^, we observed that expression states of genes closely connected to KDs were more accurately predicted to change in response to changes in KDs, when compared to non-KD genes (Supplementary Fig. 3d, f). Finally, we observed that while taking edge direction into account in validating KDs decrease the network neighborhood size, and therefore the percentage of nodes with significant enrichment given the subsequent decrease in power (Supplementary Fig. 3c, e), edge direction enables more accurate predictions for KDs (Supplementary Fig. 3d, f). This demonstrates the importance of edge directionality in increasing the specificity of our networks’ predictions.

In the AD context, local KDs of interest are those predicted to modulate components of the network enriched for gene and protein AD DE signatures. Thus, to predict AD KDs, we projected the DE signatures for each AD clinical and neuropathological trait onto each of the three BNs. Each network projection consisted of overlapping gene and protein nodes from the DE signatures with all nodes in their respective BNs. We then extracted all nodes in each network within a path length of 6 (layers) in this overlap, and identified the largest connected subgraph in the network from this set of nodes and associated edges. KDA was carried out on each subnetwork resulting from these projections, resulting in a list of 499 unique KDs at FDR < 0.05 across the three networks (Fig. 1d, Supplementary Data 3).

To assess the importance of KD genes (versus genes not predicted as KDs by our models) complementarily to perturbations in experimental systems, we examined the tolerance to genetic variations in KD versus non-KD genes. We hypothesized that, given the causal regulatory role KD genes play, they would be less tolerant to genetic variation, leading to functional changes. We therefore asked whether KD genes were more intolerant to loss-of-function (LoF) variation than other genes. Using the probability of being LoF intolerant (pLI) score^38^, we found that global KD genes identified in at least one of our discovery or replication networks (Fig. 1e) had a significantly higher pLI score than non-KD genes (one-sided Wilcoxon’s test *p* value = 2.73e − 113). We also observed that pLI score increased with the number of times a gene was identified as a KD across all networks (Fig. 4b). Thus, the number of times a gene is observed as a KD in our models is an important metric for prioritizing KDs (Fig. 4b).

Given the large number of KDs identified, we sought to prioritize KDs for further exploration. As stated above, the number of networks in which KDs appear is significantly associated with higher pLI score. Additionally, AD DE signatures enable us to define centrality to AD processes. Finally, AD discriminatory power of KDs allows one to directly assess importance to disease. We therefore prioritized AD KDs according to these metrics: their conservation across multiple networks, the number of times they were identified as KD across all networks and AD DE signatures, and their ability to distinguish AD cases from controls.

We first characterized the distribution of the number of times a gene or protein was identified as a KD across all projections (Figs. 1d and  4b). The multiscale network was of particular interest, as it integrated gene- and protein-expression data in the MSBB population (Fig. 4a). KDs identified from this single coherent network structure are depicted in Fig. 4d. Only one KD, *VGF*, was conserved across all three networks, supporting its potential importance in AD. Other KDs that appeared in multiple networks included genes known to be important for AD, including *GFAP*, *MAOB*, and *GSN* (Supplementary Table 3) serving as internal positive controls for our modeling approach.

To complement prioritizing KDs by their replication across multiple networks, we rank ordered them by their influence in classifying AD cases and controls (“Methods”). VGF was consistently identified in the top-ranked KDs not previously causally implicated in AD (gene network rank: 6, top KD *KCNN2*; protein network rank: 2, top KD HSPB1; multiscale network rank: 2, top KD HSPB1) (Supplementary Data 3).

*VGF* was the only KD identified across all AD networks and the top-ranked KD not previously causally associated with AD in the AD classifiers (ranked second overall after HSPB1^39^). We therefore pursued VGF for extensive experimental validation (Fig. 1g). Given the causal role of VGF in our AD-associated networks, and given it was the top upregulated KD gene in controls, our validation hypothesis was that overexpression of VGF would not only significantly alter the state of AD associated network components it was predicted to regulate, but that it would protect against AD.

### Replication and validation of VGF as a KD in AD

To validate our prediction of VGF’s role as a KD of AD with protective effects, we pursued three independent validation paths: (1) replication of our results in different brain regions and independent datasets; (2) association of human genetic variation derived risk for AD with *VGF* expression; and (3) prospective validation of VGF in an experimental model of AD.

VGF replicates across different brain regions and in independent datasets. To further support VGF as a KD for AD and assess its regulatory role across brain regions, we applied the same analysis pipeline (Fig. 1a–d), allowing for slight variations required for these data, to multiple brain regions in the AMP-AD MSBB dataset. We identified *VGF* as a KD in two of the three additional brain regions in the MSBB dataset, the superior temporal gyrus (BM22, Supplementary Data 5) and the pars opercularis (BM44, Supplementary Data 5). *VGF* did not reproduce as a KD in the brain region most affected by the disease (highest number of DE genes), the ectorhinal area (BM36, Supplementary Data 5), potentially reflecting a complete disruption of regulatory networks in brain regions badly damaged by AD. We applied this analysis pipeline (Fig. 1a–d) on an independent dataset, the Religious Order Study and Memory Aging Project (ROSMAP) dataset^24,25^, using DNA and RNA data generated in the same brain region as our original result, the dorsolateral prefrontal cortex (PFC). In the ROSMAP PFC network that resulted, *VGF* was identified as a KD (ROSMAP, Supplementary Data 5).

### Genetic support for VGF association to AD

DNA variations in and around VGF have not been associated with AD. As depicted in Fig. 4b, KD genes like *VGF* are much less tolerant to genetic variations leading to a functional change. Potentially related to this, we did not identify any eQTL or pQTL for VGF, although one strength of an integrative, causal network-based approach is the ability to infer causality complementarily to direct methods such as GWAS. We assessed the relationship between *VGF* expression in BM10 and the genome-wide risk for AD. We computed an AD polygenic risk score (PRS) for the European subset of our cohort^23^ (*N* = 177) from 13,704 linkage disequilibrium (LD)-independent SNPs with a *p* value for AD association below 0.0293 in I-GAP^10^ (optimal threshold determined by PRSice2^40^). These AD PRS were significantly associated with *VGF* expression in a direction consistent with our network model prediction: lower *VGF* expression associated with higher AD PRS (*p* value = 1.9e − 4, Nagelkerke’s *R*^2^ = 0.076).

### In vivo molecular and physiologic validation of VGF as an AD KD

To validate the KD role of VGF in AD pathogenesis and progression (Fig. 1f), we modulated VGF levels in the transgenic 5xFAD amyloidopathy mouse model (expressing human Presenilin1 (PS1) and APP containing five FAD mutations)^41^. 5xFAD mice were crossbred to a VGF germline homozygous knock-in mouse model (VGF^Δ/Δ^)^14^, in which insertion of a pgk-neo selection cassette into the *Vgf* 3′-untranslated region (3′-UTR) leads to a VGF messenger RNA (mRNA) truncation in the 3′-UTR region (Δ3′-UTR), resulting in increased protein translation and elevated VGF protein levels in mouse brain (Supplementary Fig. 4a).

Levels of VGF protein in VGF^Δ/Δ^ hippocampus are modulated in a similar “physiological range” (increased to ~150–200% control) as VGF protein levels are altered in male mice following chronic social defeat stress (decreased to ~50% control in the hippocampus and increased to ~140% control in the nucleus accumbens). VGF mRNA levels are similarly regulated in human control subjects and patients with major depressive disorder (MDD), being reduced in the hippocampus to ~50% control in male and female MDD patients and increased in male MDD nucleus accumbens to ~150% control^42^.

**Fig. 5:**
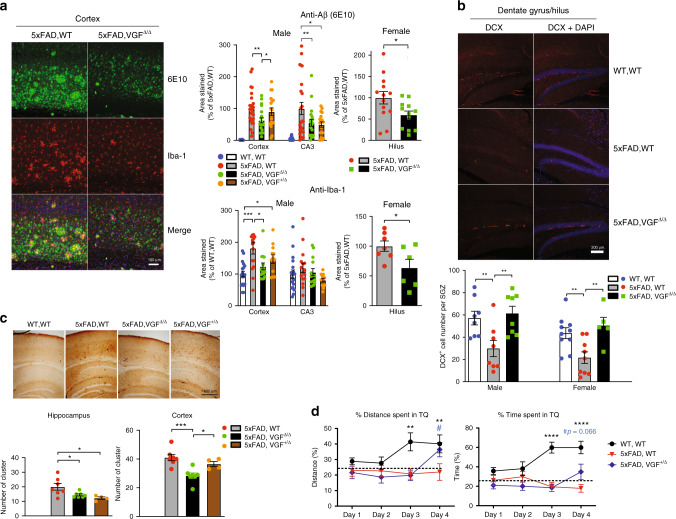
Characterization of AD pathophysiology in wild-type, 5xFAD, and 5xFAD mice overexpressing VGF. **a** Immunohistochemical staining of Aβ amyloid plaques and microglial cells in the male mouse cortex of 5xFAD mice overexpressing VGF in the germline. Left panel, green: Aβ (6E10), red: Iba-1, blue: DAPI; right panel, quantification of percent area of Aβ and Iba-1 staining in male and female mice. Quantification of percent area of Aβ and Iba-1 staining in the cerebral cortex, hippocampal CA3, and hilus; data are presented as mean percentage ± SEM of the control group. One-way ANOVA with Newman–Keuls post hoc analysis, cortex (anti-Aβ): *F*_(3,86)_ = 30.84, *p* < 0.0001, CA3 (anti-Aβ): *F*_(3,86)_ = 12.44, *p* < 0.0001, cortex (anti-Iba-1): *F*_(3,56)_ = 7.307, *p* = 0.0003, *n* = 9, 8, 7, 6 mice per group, 2–3 slices analyzed per animal, *p < 0.05, **p < 0.01, ***p < 0.001; female: *n* = 7, 6 mice per group, two-sided Student’s *t* test, *p* = 0.031 (Aβ), *p* = 0.0454 (Iba-1). **b** Doublecortin staining (DCX) of the subgranular zone (SGZ) in the dentate/hilus area of male 5xFAD brains. Upper panel, red: DCX, blue: DAPI; lower panel, average number of DCX-positive cells per subgranular zone. One-way ANOVA with Newman–Keuls post hoc analysis, male: *F*_(2, 21)_ = 6.652, *p* = 0.0058, *n* = 4, 4, 4 mice per group, 2 slices analyzed per animal; female: *F*_(2, 21)_ = 7.008, *p* = 0.0047, *n* = 10, 9, 5 mice per group. **p < 0.01 **c** Reduced staining of phosphor-Tau and dystrophic neurite clusters in 5xFAD brains with germline VGF overexpression. Upper panel: phosphor-Tau staining; lower panel: quantification results of dystrophic neurite clusters in the hippocampus and cortical area. One-way ANOVA with Newman–Keuls post hoc analysis, cortex: *F*_(2, 15)_ = 10.92, *p* = 0.0012, hippocampus: *F*_(2, 15)_ = 5.549, *p* = 0.0157, *n* = 7, 7, 4 male mice/per group. *p < 0.05, ***p < 0.001. **d** Barnes maze test. Mice were trained daily and WT mice learned the target quarter (TQ) of the hiding zone by increased distance traveled in the TQ (left panel) and increased time spent in the TQ (right panel). 5xFAD mice showed impaired spatial learning on day 4, while germline VGF overexpression (5xFAD,VGF + /Δ) partially restored memory performance. *N* = 12–14 mice (male + female) per group. Data were analyzed by two-way repeated-measures ANOVA. % of distance spent in TQ: Days (F_(3,108)_ = 3.215, *p* < 0.05) and Groups (*F*_(2,36)_ = 8.77, *p* < 0.001), and Days × Groups interaction (*F*_(6,108)_ = 1.9, *p* = 0.0873). % time spent in TQ: Days (*F*_(3,105)_ = 2.422, *p* = 0.07) and Groups (*F*_(2,35)_ = 20.01, *p* < 0.0001), and Days × Groups interaction (*F*_(6,105)_ = 4.501, *p* < 0.001). Tukey’s post hoc test. ^#^*p* < 0.05, **p < 0.01, ****p < 0.0001. All data in **b**–**d** are presented as mean percentage ± SEM.

Using VGF^Δ/Δ^ mice to increase germline *VGF* expression in this physiological range, we quantified Aβ deposition in brains of 10-month-old 5xFAD mouse by immunohistochemistry using 6E10 antibody. We found a dramatic decrease in 6E10-immunoreactive plaques in both cortical and hippocampal regions of 5xFAD,VGF^Δ/Δ^ compared to 5xFAD, while total brain transgenic APP protein levels remained unchanged (Fig. 5a, Supplementary Fig. 4d). Microglial activation in AD patients^43–46^ and increased microglial number and sometimes activation in 5xFAD^41,45,47^ have been reported, suggesting a pathological connection between amyloid deposition and neuroinflammation. The number of Iba-1-positive cells, a microglial marker, was significantly reduced in the cortex of 5xFAD,VGF^Δ/Δ^ compared to 5xFAD with normal levels of VGF (Fig. 5a). In addition, adult hippocampal neurogenesis is impaired in human subjects with AD^48^, and rapid and aggressive amyloid pathology in 5xFAD was associated with reduced neuron numbers and neurogenesis in the subgranular zone of the hippocampus^49^, which was fully rescued by VGF germline overexpression in male and female 5xFAD (Fig. 5b). Increased levels of Tau phosphorylation, observed in the clusters formed by dystrophic neurites around amyloid plaques in brains of human patients and mouse AD models^50,51^ including 5xFAD, were reduced by germline VGF overexpression in 5xFAD,VGF^Δ/Δ^ mice (Fig. 5c), while reduced levels of postsynaptic density (PSD)-associated protein PSD-95 in 5xFAD hippocampus (CA1) were significantly increased (Supplementary Fig. 5). Importantly, impaired spatial learning and memory of 5xFAD mice in the Barnes maze was partially restored by germline VGF overexpression (Fig. 5d).

**Fig. 6:**
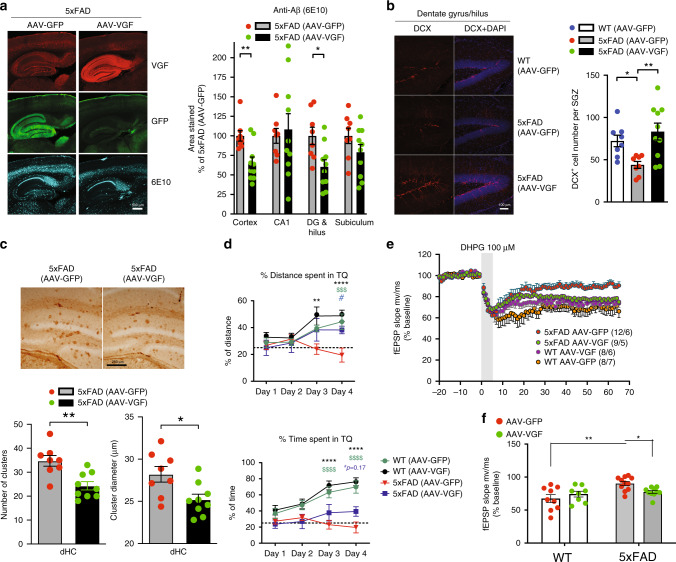
Characterization of AD pathophysiology in 5xFAD mice with and without AAV5-VGF-driven overexpression of VGF. **a** Immunohistochemical staining of Aβ amyloid plaques and VGF in the 5xFAD mouse brain 4 months after AAV5-VGF or AAV5-GFP infusion into the dorsal hippocampus. Left panel, red: VGF, cyan: Aβ, green: GFP; right panel, quantification of percent area of Aβ amyloid plaque in different brain areas. *N* = 4, 5 male mice per group. Data are presented as mean percentage ± SEM (of the control group, two-sided Student’s *t* test. **p = 0.004, *p = 0.0121. **b** Doublecortin staining (DCX) in the dentate/hilus area. Upper panel, red: DCX, blue: DAPI; lower panel, average number of DCX-positive cells per subgranular zone. *N* = 4–5 male mice per group. Data were analyzed by one-way ANOVA with Newman–Keuls post hoc analysis, *F*_(2, 23)_ = 6.574, *p* = 0.0055, *n* = 4, 4, 5 male mice per group, 2 slices analyzed per animal. *p < 0.05, **p < 0.01. **c** Reduced staining of phosphor-Tau and reduction of dystrophic neurite cluster number and diameter in 5xFAD brains with AAV5-VGF overexpression. Upper panel, phosphor-Tau staining; lower panel, quantification results of dystrophic neurite cluster number and diameter in the dorsal hippocampus. *N* = 4, 5 male mice per group. Data were analyzed by two-sided Student’s *t* test. *p = 0.0162, **p = 0.0021. **d** Barnes maze test. Mice were trained daily and on Day 4 WT mice learned the target quarter (TQ) of the hiding zone, as revealed by increased distance traveled in the TQ (left panel), and increased time spent in the TQ (right panel). 5xFAD mice with AAV5-GFP showed impaired spatial learning on day 4, while in 5xFAD with AAV5-VGF overexpression, memory performance was significantly rescued. *N* = 12, 9, 10, 7 mice (male + female) per group. Data were analyzed by two-way repeated-measures ANOVA. % of distance spent in TQ: Days (*F*_(3,102)_ = 5.000, *p* < 0.01) and Groups (*F*_(3,34)_ = 5.997, *p* < 0.01), and Days × Groups interaction (*F*_(9,102)_ = 2.371, *p* < 0.05). % time spent in TQ: Days (*F*_(3,102)_ = 11.39, *p* < 0.0001) and Groups (*F*_(3,34)_ = 13.62, *p* < 0.0001), and Days × Groups interaction (*F*_(9,102)_ = 3.824, *p* < 0.001). Tukey’s post hoc test. ^#^*p* < 0.05, **p < 0.01, ^$$$^*p* < 0.001, ****^,$$$$^*p* < 0.0001. **e** Impaired DHPG-mediated long-term depression (LTD) in 5xFAD mice is partially restored by AAV-VGF expression in the dHc. N: WT (AAV-GFP) = 8 slices from seven mice; 5xFAD (AAV-GFP) = 12 slices from six mice; WT (AAV-VGF) = 8 slices from six mice; 5xFAD (AAV-VGF) = 9 slices from five mice. **f** Summary graph of data from **e** indicating the average fEPSP slope [mV/ms (% of baseline)] during the last 5 min of recording. Data were analyzed by two-way ANOVA. Slope mV/ms (% of baseline): Genotype (*F*_(1,34)_ = 9.396, *p* < 0.001) and Groups (AAV-VGF and AAV-GFP) (*F*_(1,34)_ = 0.3282, *p* = 0.5705) and Genotype × Groups interaction (*F*_(1,34)_ = 5.045, *p* < 0.01). Newman–Keuls post hoc test. *p < 0.01, **p < 0.001. All data in **b**–**f** are presented as mean ± SEM.

To examine whether VGF overexpression in adult 5xFAD brain also reduces neuropathology resulting from Aβ overexpression, adeno-associated virus (AAV)-VGF and AAV-GFP (control) were injected into adult 5xFAD dorsal hippocampus (dHC) mice at 2–3 months of age. We chose dHc because: (i) VGF peptide administration to dHC of wild-type (WT) mice has pro-cognitive efficacy^14^; (ii) local VGF ablation in mouse dHC results in memory deficits^14^; (iii) dHc has proximity to the ectorhinal area, which sustains the most damage in AD and where VGF is significantly downregulated for multiple AD features. Mice were sacrificed for histological analysis at 7 or 10 months of age following behavioral testing. Robust VGF overexpression was transduced by AAV-VGF administration to the 5xFAD dHC (Fig. 6a). Reduced 6E10-immunoreactive plaque levels were found in the hippocampal dentate gyrus and nearby cortical regions (Fig. 6a), while overall levels of transgenic APP protein were not significantly different in AAV-VGF compared to AAV-GFP-infused 5xFAD hippocampus (Supplementary Fig. 4c). Similar to germline VGF overexpression, dHC AAV-VGF administration also restored neurogenesis in 5xFAD hippocampus to WT control levels, and significantly reduced the number of dystrophic neurite clusters in the hippocampus (Fig. 6b, c). At 10 months of age, AAV-VGF-administered 5xFAD had significantly improved spatial learning and memory performance in the Barnes maze compared to those administered AAV-GFP, while VGF overexpression in non-transgenic WT mice did not enhance memory, indicating a critical role for VGF in the pathological progression and behavioral impairment of the 5xFAD mouse model (Fig. 6d).

Impaired synaptic plasticity has been linked to hippocampus-dependent spatial memory deficits in animal models of AD^52–54^. We found that hippocampal slices from 5xFAD mice failed to produce mGluR1/5-mediated long-term depression (mGluR-LTD) (Fig. 6e, f). Importantly, hippocampal LTD has been implicated in the consolidation of long-term spatial memory^55^, and deficits in mGluR-LTD have been reported in the APP/PS1 mouse AD model^54^. Hippocampal AAV-VGF overexpression partially rescued mGluR-LTD deficits in 5xFAD hippocampal slices compared to AAV-GFP-injected mice, but had no effect on WT slice mGluR-LTD (Fig. 6e, f), while baseline synaptic function was not significantly affected (Supplementary Fig. 4f), suggesting VGF-mediated restoration of mGluR1/5-dependent synaptic plasticity may contribute to the partial rescue of spatial memory deficits in AAV-VGF-infused 5xFAD mice.

**Fig. 7:**
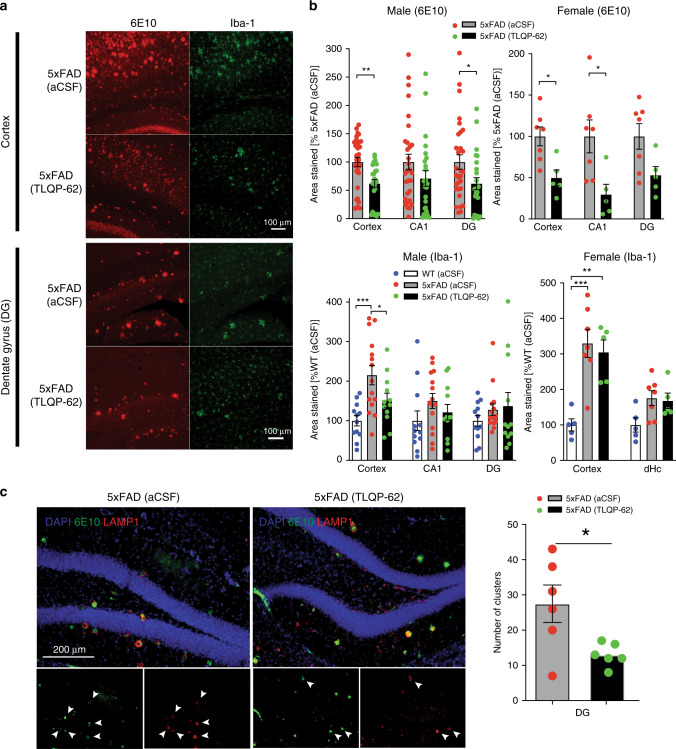
Chronic i.c.v. administration of TLQP-62 peptide ameliorated pathophysiological changes in the 5xFAD mouse brain. **a** Immunohistochemical staining of Aβ amyloid plaques and microglial cells in the male 5xFAD mouse cortex and dentate gyrus after 28-day i.c.v. administration of TLQP-62 peptide or vehicle control (aCSF). Red: Aβ (6E10), green: Iba-1. **b** Quantification of percent area of Aβ and Iba-1 staining in both peptide-treated male and female 5xFAD mouse brains. Data are presented as mean percentage ± SEM. Results of Aβ(6E10) staining were analyzed by two-sided Student’s *t* test. Male: cortex, *p* = 0.0015; DG *p* = 0.0316; female: cortex, *p* = 0.0101; CA1, *p* = 0.022. Iba-1 staining were analyzed by one-way ANOVA with Newman–Keuls post hoc analysis, male cortex: *F*_(2,36)_ = 8.449, *p* = 0.001, *n* = 4, 5, 4 mice per group, 3 slices analyzed per animal; female cortex: *F*_(2,14)_ = 12.53, *p* = 0.0008, *n* = 5, 7, 5 mice per group, *p < 0.05, **p < 0.01, ***p < 0.001. c Reduced staining of Lamp1-immunoreactive dystrophic neurite cluster number in 5xFAD brains after 28-day TLQP-62 i.c.v. infusion. Red: Lamp1, green: 6E10, blue: DAPI. *N* = 6, 6 male mice per group. Data are presented as mean ± SEM and analyzed by two-sided Student’s *t* test. *p = 0.024.

VGF is processed into bioactive peptides, including the C-terminal peptide TLQP-62 (named by the N-terminal 4 amino acids and length)^56^. TLQP-62 has pro-cognitive and antidepressant efficacy and regulates neurogenesis, both BDNF dependent when the peptide is administered intracerebroventricular (i.c.v.) or directly to rodent hippocampus^14,15,17,42,57^. We investigated whether chronic 28-day i.c.v. administration of TLQP-62 to adult 3–4-month-old 5xFAD reduced neuropathology at ~4.5 months of age. Significantly reduced levels of 6E10-immunoreactive plaques and Iba-1 immunostaining were found in hippocampal dentate gyrus and cortex from TLQP-62-treated male and female 5xFAD (Fig. 7a, b), accompanied by significantly reduced numbers of Lamp1-immunoreactive dystrophic neurite clusters in the hippocampus (Fig. 7c).

**Fig. 8:**
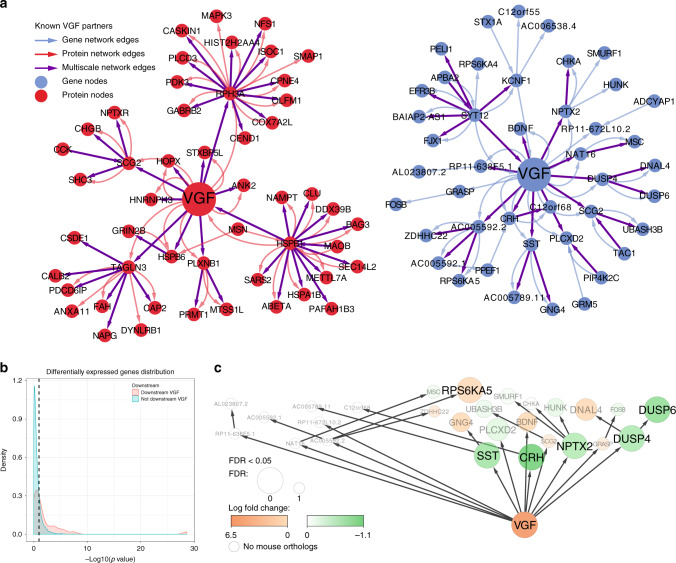
Molecular validation of VGF. **a** Consensus subnetwork within a path length of 2 of VGF. The consensus subnetworks around VGF, two steps away from VGF, across all three networks are depicted. The blue and red nodes are genes and proteins, respectively. The blue edges originate from the gene-only network, the red edges from the protein only network, and the purple edges from the multiscale network. VGF and its known partners are in bold in the plot. **b** Density plot of the distribution of differential expression nominal *p* values for genes downstream and not downstream (causally independent of the expression levels) of VGF in the gene-only network for mouse DE genes (5xFAD, AAV5-GFP versus 5xFAD, AAV5-VGF brains). The *x*-axis is the −log 10(*p* value) for differential expression, and the y-axis represents the densities at the different −log10(*p* value). The red and blue curves are for genes downstream and not downstream of VGF in the network, respectively. **c** Summary of DE results of VGF network genes in the 5xFAD, AAV5-GFP versus 5xFAD, AAV5-VGF brains overlaid on the VGF gene-only subnetwork. The nodes are colored by log fold change from green (negative) to orange (positive). The size of the node represents the DE FDR. Gray genes names are not significantly DE and white nodes have no orthologous genes in mice.

Pathophysiological validation of VGF establishes that it can induce and protect against AD-related pathologies, as predicted from our network models, but does not on its own confirm their molecular regulatory architecture. The causal networks identifying VGF provide a context that can aid in understanding mechanisms of action for genes such as *VGF*. When identifying subnetworks across all three MSBB AD BNs comprised of nodes within a path length of 2 of *VGF* (Fig. 8a), we note that Aβ and other AD genes, such as *HSPB1*, *CLU*, *MAOB*, *RPH3A*, *FOSB*, and *BDNF* (Supplementary Tables 3 and 4), are either directly connected to *VGF* or only one path length away. Additionally, other AD GWAS genes were either further downstream of VGF (PTK2B) or in its undirected vicinity (APOE, 3 path length away)^13^. To validate the molecular network, the brain gene expression signature induced by directly perturbing VGF in 5xFAD can be compared to that predicted by the network to change (Supplementary Table 1). We sequenced RNA isolated from the hippocampus of 45 mice with AAV-mediated VGF overexpression and corresponding controls, and from the prefrontal cortex of 89 mice with germline overexpression of VGF and corresponding controls. We found that genes downstream of VGF in the gene BN (predicted perturbation) were enriched for the AAV-mediated VGF overexpression DE signature (Supplementary Data 6) at a threshold of FDR < 0.05 (Fig. 8b, c, one-sided Fisher’s exact test odds ratio (OR) = 14.1, *p* value = 3.1e − 6). We also found that while the germline VGF overexpression DE signature (Supplementary Data 6) did not achieve significance at FDR < 0.05, DE genes at *p* value < 0.1 were enriched downstream of VGF in the gene BN (Supplementary Fig. 8, one-sided Fisher’s exact test OR = 3.9, *p* value = 2.6e − 3). Lastly, because BDNF is directly connected to VGF in our causal network, we assessed the impact of VGF overexpression on BDNF signaling. VGF overexpression increased BDNF receptor activation (pTrkB levels) and rescued decreased levels of pTrkB in 5xFAD brain, finding increased pTrkB levels in VGF^Δ/Δ^ mice (Supplementary Fig. 4a) and in AAV-VGF-infused 5xFAD (Supplementary Fig. 4c), compared to WT or AAV-GFP-infused 5xFAD mice, respectively. Taken together, these results validate the molecular structure of the subnetwork around VGF.

## Discussion

Our primary aim was to discover novel critical genes and pathways central to AD that could be pursued as therapeutic targets. To this end, we applied a multiscale causal network modeling approach on the AMP-AD dataset in BM10 that enabled distinguishing simple DE genes from their master regulators, whose expression changes are predicted to be causal to regulatory changes, impacting susceptibility to or protection against AD. We identified *VGF* (chromosome 7: 100,805,790–100,808,874 (GRCh37/hg19)), as a novel KD of AD. *VGF* was the most significantly downregulated gene in the protein (DE FDR = 3.4e − 15) and gene (DE FDR = 5.0e − 4) expression data in cases versus controls, and it was the only gene identified as KD across all three Bayesian causal networks constructed. Further, *VGF* ranked as the top KD having the most explanatory power in distinguishing between AD cases and controls. We replicated *VGF* as a KD in an independent dataset and in two additional brain regions, demonstrated genetic support with the association of AD PRS to the levels of expression of *VGF*, and validated *Vgf* in vivo at the physiologic and molecular levels as a KD of AD.

The biological coherence of protein expression compared to gene expression data, with respect to association with AD clinical features, was noteworthy, suggesting proteomic data may be a more informative measure for identifying important dysregulated pathways. Aβ, a hallmark of AD^6^, was consistently the protein with the highest expression in cases relative to controls, whereas VGF was the most downregulated. DE proteins are annotated for energy metabolism and immune and nervous system-related processes, all previously implicated in AD^8,58–62^; our co-expression network analyses based, in part, on these DE protein studies have further identified novel, potentially druggable targets within these pathways.

Modeling AD using BN and KDA assumes that complex genetic diseases result from dysregulated molecular networks where central hubs modulate the overall state. Here, we showed molecular validation of a large proportion of subnetworks in our models from a wide range of previously published single-gene perturbation experiments, demonstrated the added predictive value of KD nodes, and delineated the importance of edge directionality to prediction specificity. Strikingly, KDs in our networks were associated with higher intolerance to LoF variants, supporting this analytical framework for identifying molecular processes important to the health. This intolerance increased as a KD was present in more networks, emphasizing the utility of integrating multiple omics and datasets to prioritize hypotheses. These results are consistent with the recently put forth omnigenic model^63,64^, which could be tested by adapting our analytical framework and proposes that risk loci for polygenic traits converge on the regulatory networks of “core” genes. While this modeling approach enables organization of large-scale molecular data into meaningful models that can inform on disease, one limitation is that our data may not identify genes that are well known to be involved in AD (e.g., *APOE*^13^).

The *VGF* (*vgf*) gene we identified and validated is NGF and BDNF inducible and expressed in neurons in many different brain regions, encoding a 615-amino-acid (617 in mouse) precursor polypeptide that is processed into several bioactive peptides, regulating neuronal activity and survival, neurogenesis, energy balance and lipolysis, and behavior^14,15,56,65–68^. VGF is robustly regulated in the hippocampus by voluntary exercise^69^ and by BDNF/TrkB signaling^70^, and in our studies, VGF overexpression rescued cognitive deficits and neurogenesis in 5xFAD mice (Figs. 5 and 6). The VGF-derived peptide TLQP-62 regulates neuronal activity, neural progenitor proliferation, memory formation, and depression-like behavior^14–17,42^, via mechanisms largely dependent on BDNF/TrkB signaling^14,15,68,71^, and was never previously shown to be causal to AD. In addition, TLQP-21, a sub-peptide of TLQP-62, activates the complement 3a receptor (C3aR1)^66^; C3a activation of C3aR1 on microglia regulates amyloid uptake and microglial migration in primary microglia and/or mouse AD models^72^. Consistent with a role for these VGF-derived peptides in regulation of hippocampal neuronal plasticity and potentially AD pathogenesis, VGF C-terminal peptides TLQP-62 and/or TLQP-21 regulate hippocampal dendritic length and branching, synapse number, and synaptic protein levels, in vitro and/or in vivo^14,16,42,73,74^. Lastly, the VGF_1–617_ proprotein, and secretogranin 2, identified in VGF gene and protein networks, function in dense core vesicle (DCV) biogenesis and exocytosis^75^.

Trait studies have found VGF levels reduced in CSF of patients with AD^18–22,76^, decreasing with disease progression, in agreement with our findings that VGF is the gene and protein product with the lowest expression in cases relative to controls. Interestingly, reduced VGF levels were detected prospectively in CSF from patients with mild cognitive impairment, selectively in those who develop AD^20,22^. Although CSF levels of VGF, a neuronal and neurosecretory protein, might be anticipated to decrease coincident with neuronal loss as AD progresses, CSF levels of several related neurosecretory and synaptic proteins, including chromogranin A, secretogranin II, 7B2, proSAAS, clusterin, neurexins 1, 2, and 3, and neuropentraxin 1, were either increased or unchanged in patients with AD compared to controls, while VGF levels were consistently reduced^18,20^. While CSF biomarkers including VGF have utility in the diagnosis of AD, comparison of serum or plasma VGF levels in AD and control subjects has less diagnostic specificity, as plasma VGF levels are reduced in Parkinson’s disease, amyotrophic lateral sclerosis (ALS), and MDD, and are regulated by obesity and type 2 diabetes (Supplementary Table 3).

Notably, many of the genes in the VGF RNA network (Fig. 8), including VGF and BDNF, are CREB regulated^77^, and the encoded proteins modulate neuronal activity, synaptic function, and memory, are neuroprotective, and levels are reduced in AD brains (Supplementary Table 4). Moreover, VGF-derived peptide TLQP-62 activates the CREB signaling pathway, supporting VGF’s role as a KD of these CREB-responsive network genes^14,71^. Nodes within the VGF-driven protein network regulate axonal and dendritic structure, plasticity, and the trafficking and release of synaptic and DCVs, reinforcing an important homeostatic role for VGF levels in maintaining neuronal integrity (Supplementary Table 3). Relevant to our analysis, protein crosslinking studies identified a VGF interaction with amyloid precursor-like protein 1^78^, which could impact VGF or β-amyloid function in AD.

AAV-mediated VGF overexpression in 5xFAD dHc increased expression of three VGF network genes (*BDNF*, *MSK1*, and *GNG4* (G protein subunit gamma 4), Fig. 8c) as our network model predicted, all shown to play potential roles in AD^21^. BDNF in combination with increased adult hippocampal neurogenesis and exercise^79^ improves cognition in 5xFAD mice, while the BDNF Val66Met SNP modulates neuropathology and cognitive decline in subjects with AD^80^. Exercise plays a preventative role in AD and increases neurogenesis in 5xFAD^79,81,82^, and VGF and BDNF levels are upregulated by exercise in mouse models^69,79^. Consistent with these studies and our network, VGF overexpression increased levels of activated BDNF receptor (pTrkB) and adult hippocampal neurogenesis, increased levels of the BDNF/TrkB regulated postsynaptic protein PSD-95^83–85^, and improved cognition in 5xFAD (Figs. 5 and 6, Supplementary Figs. 5 and 6). Rescue of PSD-95 expression in 5xFAD would be anticipated to restore BDNF-induced TrkB/PSD-95 complex formation and TrkB signaling^86^. Also in the VGF network, mitogen- and stress-activated kinase (MSK1 or RPS6KA5) regulates BDNF signaling to CREB, hippocampal neurogenesis, synaptic plasticity, and cognition (Supplementary Table 4). Lastly, GNG4 has been implicated in cognitive decline during aging and is downregulated in aged 5xFAD mice compared to age-matched WT (Supplementary Table 4). Taken together, these studies validate the contributions of several VGF network genes to AD, and further suggest that a VGF/TLQP-62/BDNF/TrkB autoregulatory loop could function to slow or reverse neurodegeneration, much as it functions in cognition and depression^14,42,68^.

Constructing and validating AD models, which serve as integrated and comprehensive repositories of the regulatory frameworks of AD, provide a more informative and accessible path for others to leverage extensive sets of data from which they can validate links between known disease targets, generate hypotheses around novel targets, and derive mechanistic insights furthering our understanding of AD. The focus of our work was to construct a predictive model of AD and validate the top master regulator identified by the networks. Indeed, the data presented here are consistent with causal roles for TLQP-62 and the VGF proprotein in AD pathogenesis and progression, but do not rule out contributions of other VGF-derived peptides including TLQP-21, an activator of the C3aR1 complement receptor^66^. Of note, we and others have recently shown that TLQP-21 reduces neuropathology in male 5xFAD mice and increases amyloid uptake in transformed BV2 mouse microglia and in primary cultured mouse microglia via a C3aR1-dependent pathway^87–89^, consistent with the previous identification of a critical complement network module in AD^5^.

## Methods

### Methods references

All references for the “Methods” can be found in Supplementary Table 1.

### Data description

All MSBB discovery and replication datasets were previously described in Wang et al.^23^. These consists of gene and protein expression and whole-exosome sequencing (WES) for a cohort of individuals across the entire spectrum of AD in the Mount Sinai Brain Bank. Four brain regions were assessed: the anterior prefrontal cortex (BM10), the superior temporal gyrus (BM22), the perirhinal cortex (BM36), and the pars opercularis (BM44). RNA-seq was performed for 1096 samples from 315 individuals across all four brain regions, and MS/MS for 266 samples from 266 individuals in BM10 to measure protein expression. WES was performed for 309 individuals. All human research was carried out in accordance with the policies and procedures of the Icahn School of Medicine at Mount Sinai and its Institutional Review Board. For validation experiments, RNA-seq was performed for 89 5xFAD mice with and without germline overexpression of VGF and 45 5xFAD mice with and without AAV-VGF injection. Sequencing was completed following the same procedure as the human data (Wang et al.^23^) to achieve a mean coverage of 23 million reads using Illumina HiSeq 2500 System with 100 nucleotide single end reads, according to the standard manufacturer’s protocol (Illumina, San Diego, CA). The RNA for all samples was treated with Ribo-Zero (Human/Mouse/Rat) (Illumina, San Diego, CA) to remove ribosomal RNA (rRNA) and retain other transcripts. The disease was categorized in six different ways, each representing different aspects of AD: CDR, clinical neuropathology (Path Dx), CERAD neuropath criteria, neuropathology category (NP-1), mean neocortical plaque density (PlaqueMean, number of plaques/mm^2^), and bbscore. The ROSMAP^24,25^ validation set consists of gene expression from the dorsolateral prefrontal cortex of 724 subjects and whole-genome sequencing (WGS) data from 1200 subjects. The ROSMAP RNA-seq count matrix and associated quality measurements were downloaded from https://www.synapse.org/#!Synapse:syn9702085, where their generation is described. The ROSMAP WGS data variant call format (VCF) file was downloaded from https://www.synapse.org/#!Synapse:syn10901595, where its generation and quality control are described.

All data are available at https://www.synapse.org/#!Synapse:syn2580853/wiki/409853.

### RNA-seq processing

To ensure a reliable set of samples and genes for all analyses in the MSBB datasets, we performed QC processing and filtering for lowly expressed genes on the whole dataset across all four brain regions. Starting with the raw RNA-seq reads, we aligned (using STAR alignment) to GRCh37 and counted the reads mapping to each gene (featureCounts) as well as created QC matrices and called variants (using GATK) on the RNA-seq with the RAPiD pipeline^27^. For RNA-seq samples sequenced multiple times, we selected the fastq file (raw reads), which had the largest number of mapped reads and <5% rRNA mapped reads. We then ran STAR alignment and featureCounts to generate a raw count matrix and called RNA-seq variants using GATK. We also called variants on the WES using GATK. We next imputed sex information for each sample using RNA expression and variants from the WES data. By comparing the heterozygous variants from the RNA-seq data to the variants in the WES data, we were able to assign each RNA-seq sample to its corresponding DNA sequence. Using these multiple layers of information, we corrected any mislabeling, when necessary. For RNA-seq samples with documented matching WES, if the discordance rate between said sample and its best corresponding exome sequence was >10%, they were removed from further analyses. This left 958 RNA-seq samples in the MSBB dataset. In the ROSMAP gene expression data, we found one sample were gender was mislabeled and removed it from further analyses.

In the MSBB data, to filter out low expressed genes, we removed all genes that did not have at least 1 count per million (c.p.m.) in at least 10% of the samples. We normalized the raw counts using the voom function from the limma R package. After exploration of the main drivers of variance using principal component (PC) analyses and using linear mixed models (variancePartition), we adjusted the normalized counts for batch effects using linear mixed models. The corrected residuals were further adjusted with the lmFit function of the limma package for postmortem interval (PMI), race, sex, RNA integrity number (RIN), and exonic mapping rate. Sex was included as a covariate in all modeling procedures to ensure that sex-specific differences in AD were accounted for. Outlier samples further than 3 standard deviations (std) from the centroid of PC1 and PC2 were removed from downstream analyses. Samples with RIN < 4 were removed from further analyses. The raw counts of the 886 samples remaining were then subjected to the exact same protocol, to get normalized and adjusted gene expression for 24,865 genes.

From the processed MSBB data, we identified 18 samples for which variants from RNA-seq and WES data did not achieve the level of concordance expected for samples derived from the same donor. In addition, for six samples the sex inferred by DNA and RNA data did not match the sex reported for the corresponding participant in the clinical report, and 13 of the RNA-seq samples mapped to more than one WES sample (discordance rate with best matching sample >10%). We removed from all further analyses 16 of these samples that could not be unambiguously corrected (Supplementary Fig. 9a, b, Supplementary Data 1). Finally, we removed all RNA-seq measurements with RIN <4, leaving 246 samples in BM10 for detailed analyses.

To assess integrity of these data and identify covariates that could impact our analyses, we carried out variance partition and PC analyses, and identified exonic mapping rate (fraction of reads mapping to exonic regions), RIN, and sequencing batch as covariates explaining the greatest variation in gene expression across samples (Supplementary Fig. 9c–h). To minimize the impact of these covariates on detecting our primary signal of interest (association of molecular traits to AD), we adjusted the normalized RNA-seq count data for these main drivers of technical variation, in addition to race, sex, and PMI (Supplementary Fig. 9d–h) using linear mixed models. Protein-expression data were processed in a similar fashion and corrected for batch, PMI, race, and sex to minimize unwanted variation.

In the ROSMAP dataset, we followed a similar protocol removing all genes that did not have at least 1 c.p.m. in at least 10% of the samples, normalized using the voom function and after exploration of the main drivers of variance, adjusted the normalized counts for batch, sex, race, PMI, RIN, median 5′ to 3′ bias, strand balance, and percent of intronic bases using the lmFit function. The RIN values in this dataset were from 5.0 to 9.9 (mean = 7.06, std = 0.99). The output was a matrix of normalized and adjusted counts of 19,452 genes for 633 samples.

The mouse RNA-seq data was aligned to the mouse genome (GRCm38), version M10 (Ensembl 85) following the same procedure as described for the human data above. For both overexpression models (AAV-VGF and VGF^Δ/Δ^), no covariates were found to drive variance of the data, and normalized counts were directly used for DE analyses.

### Protein-expression processing and correction for other covariates

The protein-expression data was taken through similar procedures to ensure that technical variation was accounted for. After correction for batch on the protein-expression data for 266 samples, we further adjusted for PMI, race, and sex using the lmFit function of the limma package. The remaining 2692 protein-expression residuals were used for downstream analyses.

### DE analyses

The DE analyses were performed for both gene and protein expression using the limma package after the adjustment for covariates described earlier. To capture all aspects of the disease, the DE was performed for each AD trait. In addition, to capture signal corresponding to the entire spectrum of AD, DE analyses were performed in two ways: controls against any sample that had any level of cognitive impairment (and in the case of PlaqueMean using its quantitative level as a response), and definite controls against definite AD as defined by each trait (Supplementary Table 2). Validation of DE was done with gene set enrichment analyses R packages GOtest and msigdb. The public DE sets were assembled from the literature derived significantly differentially expressed genes and proteins. The GWAS set is comprised of the genes associated to AD in the latest AD GWAS^13^ and the GWAS in TAD set is comprised of all the genes within topologically associated domains containing a significant locus (defined as the lead SNP and the SNPs with *R*^2^ > 0.5 with them). *P* values were adjusted for multi-testing using Bonferroni correction.

### QTL analyses

All QTL analyses were run using the fastQTL package. Using plink2, we removed markers with >5% missing rate, <1% major allele frequency, and Hardy–Weinberg *p* value <10^−6^ from the WES (MSBB) or WGS (ROSMAP) variants. Following standard practices, only European individuals were used to find QTLs. Non-European samples were identified through PCA analyses using smartPCA and mapping in PC space to the 1000 Genomes Project consortium. VCF liftover was used to lift over the ROSMAP WGS from hg19 to hg38. The residuals described above were used for QTL analyses for both gene and protein expression after further correction for PEER surrogate (latent) variables (SVs) as follows: (i) BM10 gene expression: 19 SVs; (ii) BM10 protein expression: 9 SVs; (iii) BM22 gene expression: 20 SVs; (iv) BM36 gene expression: 17 SVs; (v) BM44 gene expression: 17 SVs; (vi) ROSMAP gene expression: 25 SVs. We also included in the model the first 5 PCs of the genotype data to remove further population-specific structures. The analyses looked for *cis*-eQTLs as defined 1 Mb of the transcription start site of each gene and protein corresponding gene. FDR were computed following Benjamini–Hochberg procedure. The causal inference testing was performed with the R package citpp (https://bitbucket.org/account/signin/?next=/multiscale/citpp).

### Co-expression analyses

Three WGCNA co-expression networks were built on the adjusted data, one for the gene expression, one for the protein expression, and one for both gene and protein expression (multiscale), using the coexpp R package.To identify modules of interest in the context of AD, we projected the union of all DE genes or proteins on the corresponding co-expression network. We calculated enrichment statistics using Fisher’s exact test, and corrected for multi-testing following the Bonferroni procedure.

### Seeding gene list construction

Making the assumption that DE genes are important for AD, and that therefore these genes need to be included in the model, we added the union of all DE genes to the seeding gene list. To include other important genes that covary with these DE genes, but that may not reach significance in the DE analyses, we included all genes in co-expression modules enriched for DE genes. Finally, for the discovery gene expression set only (BM10), to maximize the chances to not miss important genes, we used PEXA^34^ to add other genes known to be connected to our current gene list in the literature to the gene list of DE genes and modules of interest. To build the extended network, PEXA used KEGG pathways, and to trim it, used a PPI network from CPDB. We used the outputted discovery seeding gene list of 5714 genes for which we have gene expression for Bayesian causal network construction. For the replication sets, the seeding gene lists were comprised of only the DE genes and the co-expression modules enriched for the DE genes, and consisted of 10,585 genes for BM22, 16,578 genes for BM36, 8086 genes for BM44, and 9682 genes for ROSMAP. For BM44, due to the small number of DE genes and to increase the number of genes in the AD DE gene set with genes related to DE genes and find co-expression module enrichments, we added the top 10 correlated genes to each of the DE genes before module enrichments.

### Bayesian causal networks

BNs were built using RIMBANET using gene expression, protein expression, and both gene and protein expression (multiscale) for the discovery datasets, and using gene expression for the replication datasets. In each case, QTLs were used as priors (eQTLs for gene networks, pQTLs for the protein network, and both for the multiscale network), which both reduce the search space size and enhance causal inference among nodes^31,33^. To reduce the search space and increase the likelihood to reach a global maximum of the fit of the network, we reduced the gene space from entire expressed transcriptome (24,865 genes) to the seeding gene list described earlier for the gene-only and the multiscale networks. Since there was protein expression for only 2692 proteins, we included all the proteins in both the protein and the multiscale networks. To account for the central dogma of biology where RNA is translated into corresponding proteins and the results of the CIT analysis described earlier, we included weak edge priors (increasing the likelihood for that edge to be searched) to the multiscale network from the parent gene to its corresponding protein product. While including such guiding structure priors can result in more accurate networks, such priors are not absolute, and any such edge must ultimately be supported by the data (an important feature given our finding from causal mediation analyses that did not always support causal relationships between gene- and protein-expression traits).

Representation of networks and subnetworks was achieved using the Cytoscape software version 3.5.1.

### Key driver analyses

For both the discovery and the replication datasets, KDA was performed using the R package KDA. This package defines a background subnetwork by looking for a neighborhood *K*-steps away from each node in the target gene list in the network. Stemming from each node in this subnetwork, it assesses the enrichment in its *k*-step (*k* varies from 1 to *K*) downstream neighborhood for the target gene list. In this analysis, we used *K* = 6. KD analyses were performed by projecting multiple seeding target lists of interest on the networks: The DE lists of the corresponding omics for each disease trait to find KDs of the diseases. In the discovery dataset, KDs were then prioritized by first how many networks they appear in (replication) and then how many times they appear across networks (importance). In the replication datasets, we looked for presence of VGF as a KD of the networks.

### Validation of KD predictive power with Enrichr signatures

A matrix of distances between every node in our discovery BN models was computed with the distances function of the igraph R package. Subnetworks around each node in the networks were defined both in an undirected and downstream fashion. For each node with a perturbation in the CNS or the immune system, enrichment for signatures in subnetworks from path length 1 to 6 were computed with the R base function fisher.test and adjusted for multi-testing using the FDR setting of the p.adjust function. The proportion of nodes with significant enrichment was defined at each path length as the proportion of nodes with existing perturbation and FDR < 0.05 divided by the total number of nodes with existing perturbation. The ratio of KDs to non-KDs with significant enrichment was defined as the proportion of KDs with significant enrichments divided by the proportion of non-KDs with significant enrichments.

### Ranking of KDs using machine learning

For each classifier we performed a random split of the data, stratified by class, into 75% training set and 25% validation set. The training set was subjected to SMOTE to resolve any class imbalance for training the random forest (RF) classifier (python sklearn package). Classifier performance was evaluated against the validation set and quantified using area under the curve (AUC) of the receiver operating characteristic (ROC) curve. RF randomly subsets the features into decision trees, selecting a feature from each subset that best separates the data into classes. Therefore, the choice of a feature to be included in the forest is an indication of the performance and stability of that feature. Features were ranked by importance, as based on information gain score. This process was performed 500 times to estimate the distribution of feature information gain across classifiers. Features were then organized into a meta-rank by a weighted *z*-score method across the 500 iterations per classifier. There, a *z*-score was established from the features rank per iteration of the information gain and weighted by a factor accounting for stability of features and performance of the classifier. The weight is the product of two components: (i) the ratio of the number of iterations each feature appeared in to the mean number of all features’ iteration appearances, both across the 500 forests; (ii) the absolute value of the ROC AUC score of each classifier centered at 0, minimizing the impact of random classifiers.

The classifiers were run independently, after normalization and adjustment for covariates, on each scale of expression data: the gene expression data, the protein-expression data and the gene and protein-expression data together. In each case, the 11 AD traits were used as classes to train and test the classifiers, and a meta-rank for each feature across the 11 traits was computed using the weighted *z*-score approach across all 5500 classifiers. To further prioritize the network KDs, all KDs were ordered according to the meta-rank of features across traits for their corresponding scale of data (Supplementary Data 3).

### PRS analyses

We assessed the relationship between *VGF* expression and genome-wide risk for AD. WGS was performed as described previously^23^. SNPs with data missing in >2% of the sample, minor allele frequency <1%, or deviation from Hardy–Weinberg equilibrium (*p* < 5 × 10^−5^) were removed using Plink2. After these initial QC checks, SNPs were pruned based on linkage disequilibrium (window size = 100, window shift = 50 SNPs, VIF threshold = 2), and multidimensional scaling (MDS) analysis on the *N* × *N* matrix of genome-wide IBS pairwise distances (performed using Plink). The first five MDS components were utilized as ancestry covariates. Only individuals of European descent were included in this analysis, as described above in the QTL analysis section. Only SNPs present in the HRC (Haplotype Reference Consortium) reference set were considered. Using I-GAP AD GWAS summary statistics^10^ as discovery, AD PRS were calculated for AMP-AD individuals using PRSice2, with *VGF* expression as the response phenotype.

### Other statistical analyses

R version 3.3.1 was used for statistical analyses, unless specified otherwise. GO annotations enrichment was tested with the R packages goseq, topGO, and org.Hs.eg.db. To test MSigDB pathway enrichment, the R packages HTSanalyzeR, GSEABase, and gage were used. Figures where generated using the R packages ggplot2, scales, reshape2 (http://www.jstatsoft.org/v21/i12/), and grid. UpsetR plots were generated with the UpSetR R package. Heatmaps were produced with the function heatmap.2 of the R package gplots. Venn diagram was drawn using the VennDiagram R package. Circos (circular) plot of DE enrichments in modules were plotted using the NetWeaver R package. Canonical correlation analyses were performed with the canCorPairs function of the variancePartition R package and canonical correlation p-values were computed with the p.perm function of the CCP R package with 10,000 random sampling of the labels. Large tables were read-in and written using the R package data.table.

### Animal models and stereotaxic surgery

The generation of 5xFAD mice was described previously^41^. These transgenic mice overexpress both human APP (695) harboring the Swedish (K670N, M671L), Florida (I716V), and London (V717I) FAD mutations and PS1 harboring the two FAD mutations M146L and L286V. Expression of both *trans* genes is regulated by neuronal-specific elements of the mouse *Thy1* promoter. The 5xFAD strain (B6/SJL genetic background) was maintained by crossing hemizygous transgenic mice with B6/SJL F1 breeders. The floxed VGF mouse line was generated as recently described^65^. Homozygous floxed VGF mice that overexpress VGF mRNA and protein by virtue of the placement of the pgk-neo cassette in the 3′-UTR region of the *Vgf* gene. This leads to premature mRNA termination and polyadenylation utilizing a cryptic poly-A addition site in the inverted pgk-neo cassette, truncating part of the 3′-UTR sequence, and resulting in increased CNS expression of VGF (Supplementary Fig. 4). All mouse studies were conducted in accordance with the US National Institutes of Health Guidelines for the Care and Use of Experimental Animals, using protocols approved by the Institutional Animal Care and Use Committee of the Icahn School of Medicine at Mount Sinai.

Mice at 2–3 months of age were anesthetized with a mixture of ketamine (100 mg/kg) and xylazine (10 mg/kg). Thirty-three gauge syringe needles (Hamilton, Reno, NV) were used to bilaterally infuse 1.0 μl of AAV virus into mouse dHc (anterior-posterior (AP) = −2.0, medial-lateral (ML) = ± 1.5, and dorsoventral (DV) = −2.0 from Bregma (mm)) at a rate of 0.2 μl per min and the needle remained in place for 5 min before removal to prevent backflow. AAV5-GFP and AAV5-VGF (mouse VGF complementary DNA (cDNA)) were prepared by the Vector Core at the University of North Carolina at Chapel Hill. AAV-injected mice were used at 7–8 months of age for immunohistochemical analysis or at 10 months of age for behavioral analysis. Additional mice at 3 months of age were anesthetized with ketamine/xylazine and a cannula was implanted in the lateral ventricle (AP = − 0.1, ML = ± 1.0 and DV: − 3.0 from bregma (mm)). TLQP-62 (2.5 mg/ml) dissolved in artificial cerebrospinal fluid (aCSF) or aCSF alone was delivered i.c.v. by microosmotic pump (Alzet delivering 0.25 µl/h or 15 µg per day) for 28 days. Mice were used for immunohistochemical analysis at 4.5 months of age.

### Immunohistochemical and biochemical analysis

Immunohistochemical and biochemical characterization were performed as previously described. For biochemical analysis, to prepare total homogenate, mouse brain tissues were homogenized in ice-cold protein lysis buffer containing 50 mM Tris-HCl (pH 7.5), 140 mM NaCl, 1% Triton X-100, 0.5% Na deoxycholate, 0.1% sodium dodecyl sulfate, and 2 mM EDTA with 1× Halt Protease and Phosphatase Inhibitor Cocktail (Thermo Fisher Scientific, Waltham, MA). For immunohistochemistry, 50-μm-thick sagittal sections were incubated with the following antibodies: rabbit anti-Iba-1 (1:500; Wako, Richmond, VA), mouse anti-6E10 (1:1000; Covance, Princeton, NJ), rabbit anti-doublecortin (1:500, Cell signaling Technology, MA), rabbit anti-PSD-95 (1:500, Cell signaling Technology, MA). Sections were then incubated with appropriate secondary antibodies: anti-mouse Alexa Fluor 488 (1:500; Invitrogen, Carlsbad, CA) and anti-rabbit Alexa Fluor 594 (1:500; Invitrogen, Carlsbad, CA). For nonfluorescent immunostaining, endogenous peroxidase was quenched with phosphate-buffered saline containing 3% hydrogen peroxide, followed by amplification using the ABC system (VECTASTAIN Elite ABC HRP Kit, Vector Laboratories, Burlingame, CA). Horseradish peroxidase conjugate and 3,3′-diaminobenzidine were then used according to the manufacturer’s manual (Vector DAB, Vector Laboratories, Burlingame, CA). ThioflavinS (Sigma-Aldrich, T1892, 1% w/v stock solution) was used for labeling amyloid deposits. For immunoblotting, membranes were incubated with either anti-VGF C terminal (1:1000; rabbit polyclonal), anti-6E10 antibody (1:1000; Covance, Princeton, NJ), rabbit anti-PSD-95 (1:500, Cell signaling Technology, MA), and anti-actin (1:1000; Sigma-Aldrich) antibodies. The membranes were washed, incubated with a secondary horseradish peroxidase-labeled donkey anti-rabbit or donkey anti-mouse antibody (1/6000; GE Healthcare) for 1 h, washed again, and incubated with ECL detection reagents (Millipore). Densitometric analysis was performed using the ImageJ software.

### RNA extraction and real-time reverse transcription quantitative PCR analysis

RNA from mouse tissue specimens, obtained by dissection of the prefrontal cortex, was extracted using miRNeasy Mini Kit (Qiagen) according to the manufacturer’s protocol, and 0.25 µg was reverse transcribed using iScript reverse transcription supermix for RT-qPCR Kit (Bio-Rad, Hercules, CA). One nanogram of first-strand cDNA was subjected to PCR amplification using a SYBR green real-time reverse transcription PCR master mix (PerfeCTa SYBR Green FastMix, Quanta Biosciences). ΔΔCt method was used to quantify relative gene expression and normalized to glyceraldehyde 3-phosphate dehydrogenase.

### Behavioral testing and analysis

The Barnes maze test was performed using a standard apparatus. Ten-month-old mice were transported from their cage to the center of the platform via a closed starting chamber where they remained for 10 s prior to exploring the maze for 3 min. Mice failing to enter the escape box within 3 min were guided to the escape box by the experimenter, and the latency was recorded as 180 s. Mice were allowed to remain in the escape box for 1 min before the next trial. Two trials per day during 4 consecutive days were performed. The platform and the escape box were wiped with 70% ethanol after each trial to eliminate the use of olfactory cues to locate the target hole. All trials were recorded by video camera and analyzed with ANY-maze video tracking software (Stoelting Co., Wood Dale, USA).

### Field electrophysiology

Coronal brain slices containing the hippocampal formation were prepared as previously described. Animals were anesthetized with isoflurane and brains were rapidly removed from the skull and placed in an ice-cold modified aCSF solution containing: 215 mM sucrose, 2.5 mM KCl, 1.6 mM NaH_2_PO_4_, 4 mM MgSO_4_, 1 mM CaCl_2_, 4 mM MgCl_2_, 20 mM glucose, 26 mM NaHCO_3_ (pH = 7.4, equilibrated with 95% O_2_ and 5% CO_2_). Coronal brain slices (400 µm thick) were prepared with a Vibratome VT1000S (Leica Microsystems, Germany) and then incubated at room temperature for ≥2 h in a physiologic aCSF, containing: 120 mM NaCl, 3.3 mM KCl, 1.2 mM Na_2_HPO_4_, 26 mM NaHCO_3_, 1.3 mM MgSO_4_, 1.8 mM CaCl_2_, and 11 mM gucose (pH = 7.4 equilibrated with 95% O_2_ and 5% CO_2_). The hemi-slices were transferred to a recording chamber perfused with aCSF at a flow rate of ~2 ml/min using a peristaltic pump; experiments were performed at 28.0 ± 0.1 °C. Recordings were acquired with a GeneClamp 500B amplifier (Axon Instruments) and Digidata 1440 A (Molecular Devices). All signals were low pass filtered at 2 kHz and digitized at 10 kHz. For extracellular field recordings (field excitatory postsynaptic potential (fEPSP) recordings), a patch-type pipette was fabricated on a micropipette puller (Sutter Instruments, Novato, CA, USA), filled with aCSF, and placed in the middle third of stratum radiatum in area CA1. fEPSPs were evoked by activating Shaffers collaterals with a Concentric Bipolar Electrode stimulator (FHC 1201 Main St, Bowdoin, ME, USA) placed in the middle third of stratum radiatum 150–200 µm away from the recording pipette. Square-wave current pulses (60 ms pulse width) were delivered through a stimulus isolator (Isoflex, AMPI). Input–output curves were generated by a series of stimuli in 0.1 mA steps. LTD was induced by treating the slices with (*RS*)-dihydroxyphenylglycine (DHPG), a selective mGluR1/5 agonist, at 100 µM concentration for 5 min after 20 min of stable baseline recordings. fEPSP responses were collected for at least 60 min during DHPG washout. Analysis of the average slope (mV/ms) during the final 5 min of LTD recording was used for statistical analysis.

### Reporting summary

Further information on research design is available in the Nature Research Reporting Summary linked to this article.

## Supplementary information

Supplementary Information

Reporting Summary

Description of Additional Supplementary Files

Supplementary Data 1

Supplementary Data 2

Supplementary Data 3

Supplementary Data 4

Supplementary Data 5

Supplementary Data 6

## Data Availability

All data, methods, and materials are available either in the main text, Methods, or Supplementary information, or via the AD Knowledge Portal (https://adknowledgeportal.synapse.org). The Mount Sinai Brain Bank (MSBB) study data is available at (https://adknowledgeportal.synapse.org/Explore/Studies/DetailsPage?Study=syn3159438) via the AD Knowledge Portal (https://adknowledgeportal.synapse.org). The Religious Orders Study and Memory and Aging Project (ROSMAP) study data is available at (https://adknowledgeportal.synapse.org/Explore/Studies/DetailsPage?Study=syn3219045) via the AD Knowledge Portal (https://adknowledgeportal.synapse.org). The AD Knowledge Portal is a platform for accessing data, analyses, and tools generated by the Accelerating Medicines Partnership (AMP-AD) Target Discovery Program and other National Institute on Aging (NIA)-supported programs to enable open-science practices and accelerate translational learning. Data is available for general research use according to the following requirements for data access and data attribution (https://adknowledgeportal.synapse.org/#/DataAccess/Instructions). Source data are provided with this paper.

## Source data

Source data

## Code availability

Only standard published tools were used for the analyses presented in this manuscript.
