# Supplementary material for: Multiscale causal networks identify VGF as a key regulator of Alzheimer’s disease
Source: Nat Commun. 2020 Aug 7;11:3942. doi: 10.1038/s41467-020-17405-z (PMC7414858; doi:10.1038/s41467-020-17405-z)
Supplement: Supplementary file 1 — Supplementary Information [file 41467_2020_17405_MOESM1_ESM.pdf]

Supplementary Information

Multiscale causal networks identify VGF as a key regulator of Alzheimer's disease

Noam D. Beckmann et al.

Supplementary Strategy Overview

As described in the main text, we employed a probabilistic causal network framework to construct predictive models of AD. The input data to construct these models were generated as part of the AMP-AD consortium, and included whole exome sequencing (WES), RNA sequencing (RNA-seq, referred to as gene-expression hereafter), and protein-expression data from the anterior prefrontal cortex (Brodmann area 10, BM10) in a large cohort of post-mortem samples from the Mount Sinai Brain Bank (MSBB, N=364, with 307 having both DNA and RNA, 217 both DNA and protein, and 217 having all three), across the complete spectrum of AD clinical and neuropathological traits (from controls to neuropathologically-proven AD, Fig. 1a) and with no other co-morbidities[1]. To focus the input of molecular traits for network reconstruction on traits associated with AD, we examined associations between the molecular data and AD clinical and neuropathological features to identify AD gene- and protein-expression signatures. Gene- and protein- expression traits co-regulated with these AD signatures were found by constructing gene and protein co-expression networks. From these networks we identified highly interconnected sets of co-regulated genes (modules) that were significantly enriched for the AD signatures and for pathways previously implicated in AD (Fig. 1b). To obtain a final set of genes for input into the causal network reconstructions, we combined genes in the AD signatures and genes in the co-expression network modules enriched for these signatures (referred to here as the seed set). We further expanded this seed set by incorporating prior pathway knowledge from the literature to ensure inclusion of important AD genes potentially missed due differential expression analyses lack of power (Fig. 1b).

With our AD-centered input set of genes for network reconstructions defined, we mapped gene and protein quantitative trait loci (eQTLs and pQTLs, respectively) for expression traits in this set to incorporate QTLs as structure priors in the network reconstructions, given they provide a systematic perturbation source that can boost power to infer causal relationships (Fig. 1c) (Supplementary Table 1). The input gene set and eQTL/pQTL data from MSBB served as input into RIMBANET to construct probabilistic causal networks of AD (Fig. 1d). An artificial intelligence algorithm to detect KD genes from these network structures was then applied to identify and prioritize causal regulators of AD networks (Fig. 1d). To validate our findings, three independent approaches were employed: 1) Replication in other brain regions and independent datasets (Fig. 1e); 2) Association of human genetic risk for AD and expression of KD genes (Fig. 1f); and 3) For the top causal regulator, VGF, functional and molecular experimental validation in the 5xFAD mouse model (Fig. 1g).

Supplementary Results

Co-expression networks partition RNA and Protein expression traits into separate modules. The construction of co-expression networks from combining gene- and protein-expression traits resulted in modules comprised nearly exclusively of one type of data (either gene or protein expression) (Supplementary Data 3). While technical components of variation specific to technologies used to score gene- and protein-expression will partly explain this pattern of co-expression, given traits of a particular type are more correlated to traits of that same type than traits of other types, the complementarity of gene- and protein-expression plays a role as well. For example, while RNA measures generally reflect expression levels in cells local to the brain region assayed, select RNAs or RNA isoforms that are known to be transported into dendrites (e.g. BDNF long 3' UTR mRNA) could potentially contribute to this signal as well[2-4]. Similarly, protein measures may reflect proteins synthesized in the local brain region that was profiled, proteins that are transported in secretory vesicles via neural pathways from cell bodies in distal regions, and proteins that are locally translated from mRNAs transported from distal regions. Thus, simultaneous sampling of RNA and protein expression in a specific brain region provides complementary data sets that not only reflect linear DNA to RNA to protein synthesis, but that also capture dynamic changes in the flux of transported proteins and RNAs into the local region.

Supplementary Figures

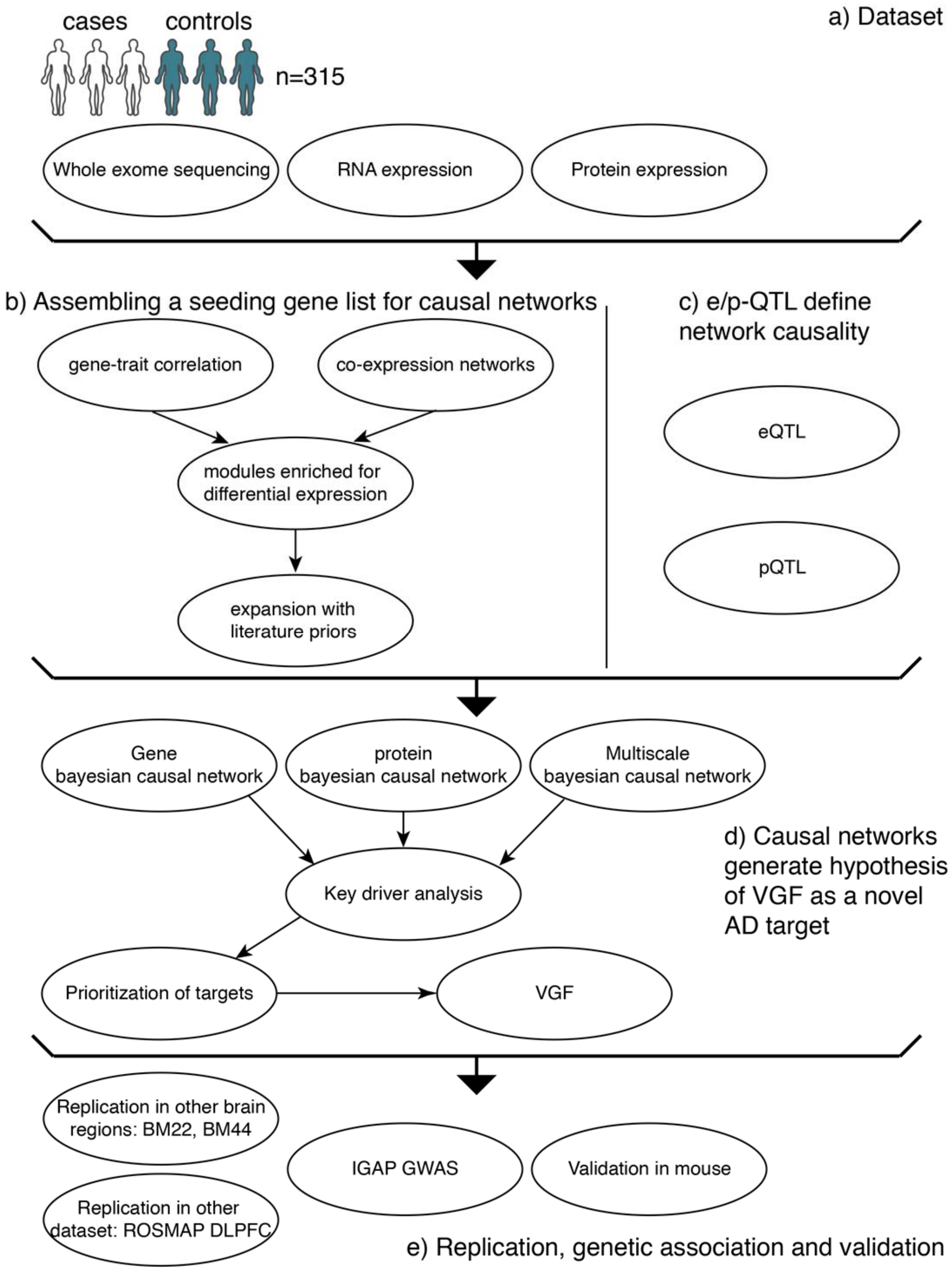

Supplementary Fig. 1.

Simplified Pipeline Overview. Simplified description of data and analyses workflows performed to identify and validate VGF as a target of AD.

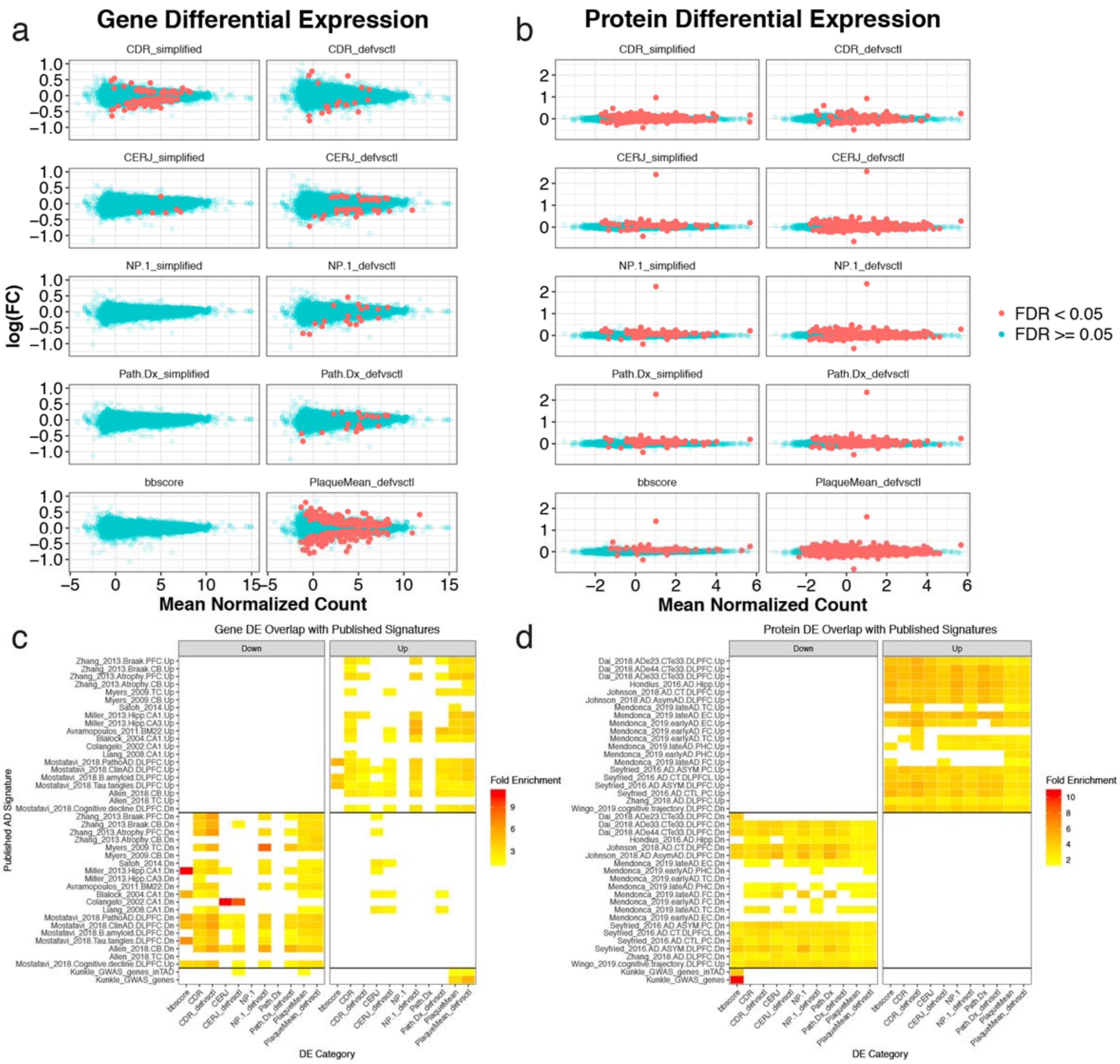

Supplementary Fig. 2.

Other AD traits gene and protein DE. a and b Gene **a** and protein **b** differential expression: The x axis of this plot is the mean normalized count for each gene or protein, and the y axis the log(FC). In blue are the non-significantly DE genes or proteins and in red the significant ones. Each box corresponds to a trait. **c and d** Heatmaps of gene **c** and protein **d** differential expression gene set enrichment analysis for published differential expression signatures, AD GWAS mapped genes, and genes in topologically associated domains containing AD GWAS loci (defined as R^2 >0.5 from lead SNP). The x axes of these plots represent the DE signatures in our dataset and in the y axes are the public DE signatures. Genes and proteins were included in the analysis if they had association to AD traits with FDR < 0.25. Heatmap shows the fold enrichment (yellow to red) for only the significantly (Bonferroni adjusted p-value < 0.05) enriched public AD signatures.

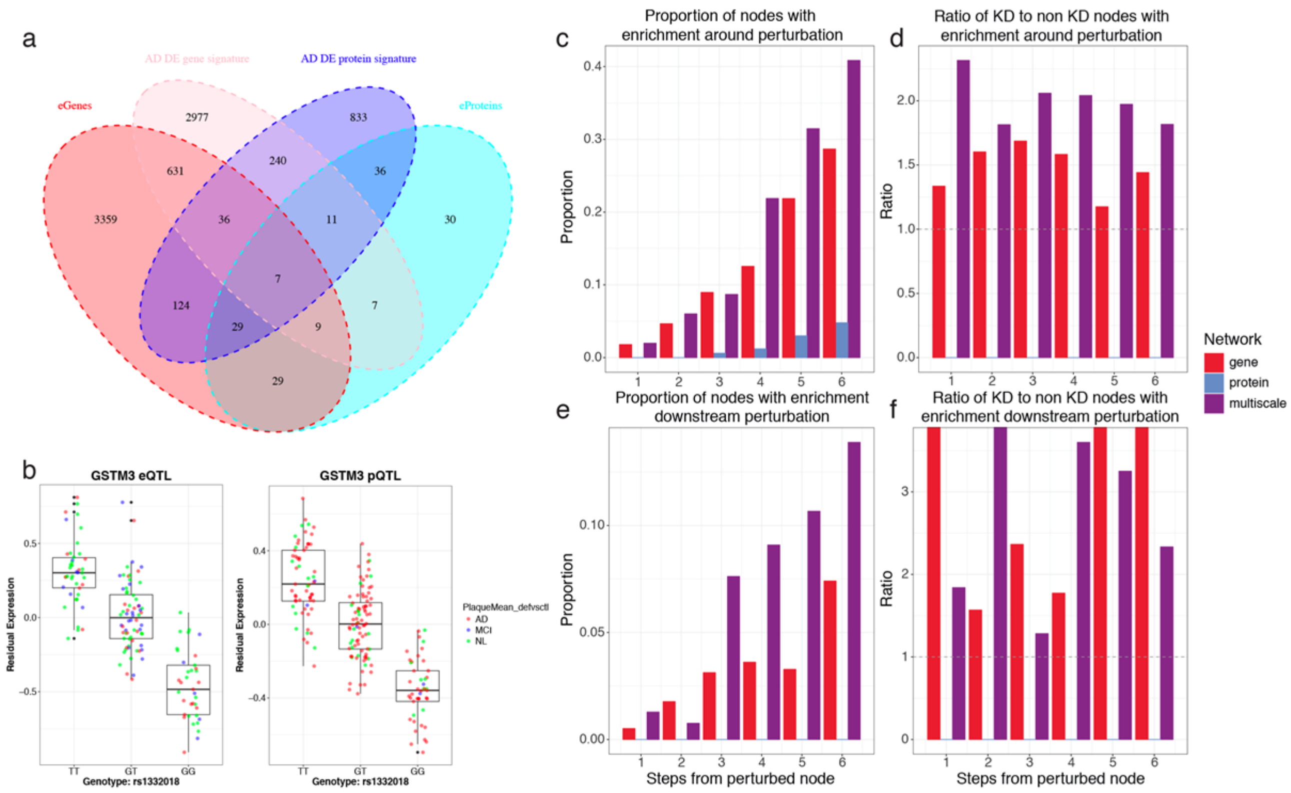

Supplementary Fig. 3.

QTL analyses and molecular validation of BN subnetworks. a Venn diagram of QTL overlap with expanded DE signatures. **b** Boxplots of QTL effects: GSTM3, gene that shares an eQTL and a pQTL at the same SNP position. **c, d, e and f** Enrichment of 742 unique perturbation (341 unique genes) signatures onto networks. In each case, the color represents the network in which the analysis is performed. **a and c** Proportion of nodes with existing signature and significant enrichment for said signature in network neighborhood **a** undirected enrichment - and **c** downstream enrichment. The x axis is the number of steps away from the perturbed node and the y axis the proportion of nodes with significant enrichment at FDR<0.05. **b and d** Ratio of global KDs with significant enrichment to non-KDs with significant enrichment in network neighborhood **b** undirected enrichment and **d** downstream enrichment. The x axis is the number of steps away from the perturbed node and the y axis the ratio of proportions of KDs with significant enrichment at FDR<0.05 to proportion of non-KDs with significant enrichment at FDR<0.05. Bars reaching top of plot indicate 0 significant non-KD enrichment.

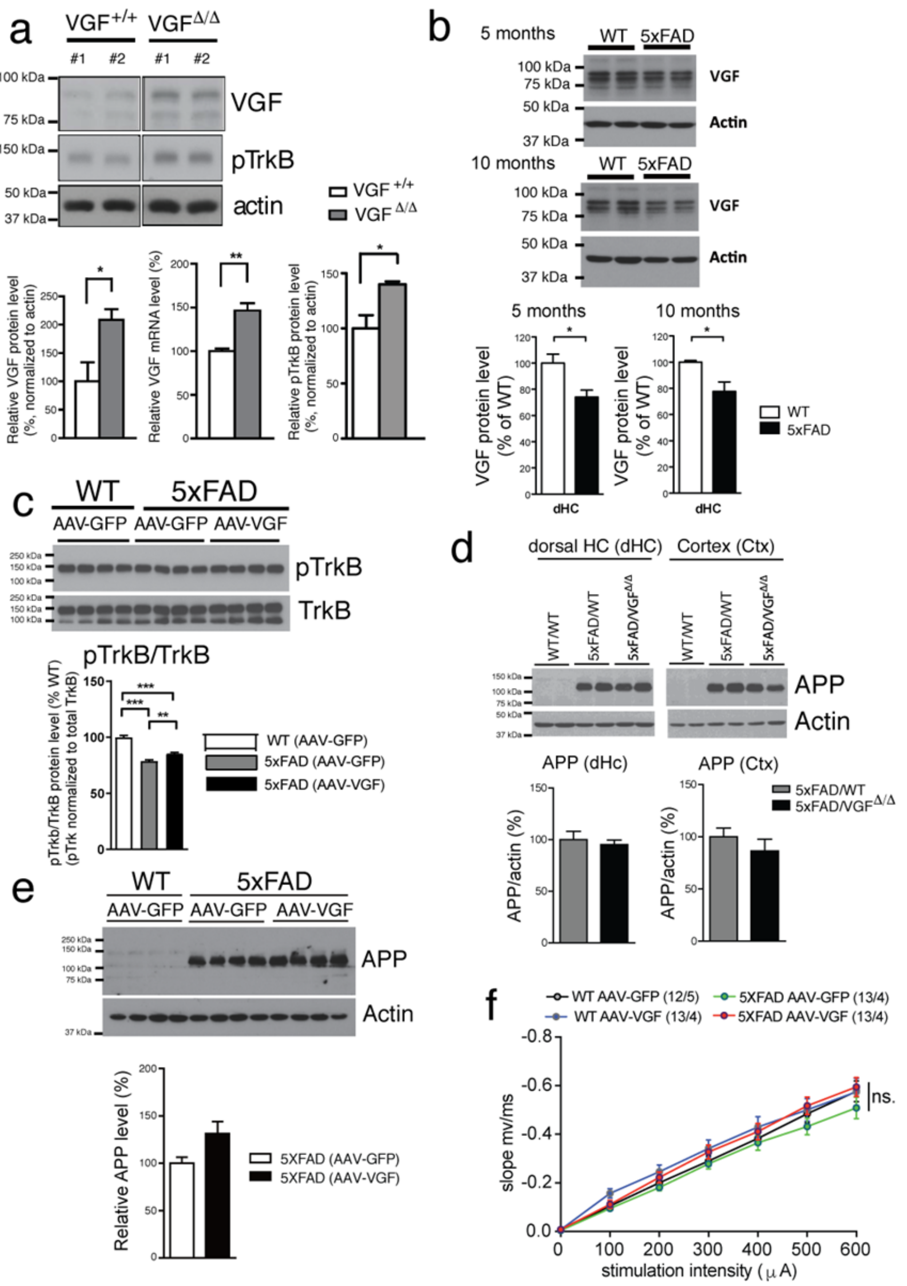

Supplementary Fig. 4.

Levels of APP expression and baseline synaptic function in WT and 5xFAD mice with VGF overexpression. a Increased VGF expression in the brains of VGF germline overexpression mouse line (VGF$^{\Delta/\Delta}$). Western blot and quantitative PCR analysis showed both increased VGF protein and mRNA level, and increased pTrkB levels, in the dorsal hippocampus of VGF germline overexpression mice (VGF$^{\Delta/\Delta}$) compared to WT (N=3 mice per group, hippocampus VGF protein: VGF$^{\Delta/\Delta}$: 208.6±18.4%; WT: 100.0±33.7%; hippocampus Vgf mRNA: VGF$^{\Delta/\Delta}$: 146.6±8.4%; WT: 100.1±3.0%, hippocampus pTrkB protein: VGF$^{\Delta/\Delta}$: 139.9±2.7%; WT: 100.0±11.9%; Student t-test, *, $p<0.05$; **, $p<0.01$. **b** VGF protein levels are reduced in the dorsal hippocampus of 5xFAD mice, at 5 months and 10 months of age. Dorsal hippocampus of

male 5xFAD and WT control mice was collected and analyzed by western blotting for VGF protein (migrating as a characteristic doublet of ~90kd). dHC, dorsal hippocampus. 5-month old, N=4~5 mice per group; 10-month old, N=4 mice per group. Hippocampal VGF protein levels: 5-month, WT: 100.0 ± 6.8%; 5xFAD: 74.1 ± 5.1%; 10-month, WT: 100.0 ± 1.2%; 5xFAD: 77.8 ± 7.1% Student t-test, *, p<0.05. **c** Partial rescue of pTrkB/TrkB levels in the brains of 5xFAD mice overexpressing VGF. Dorsal hippocampus of male 5xFAD and WT control mice was infected with AAV-VGF or AAV-GFP at ~2-3 months of age, and brain lysates were analyzed for phospho-TrkB (pTrkB) and total TrkB at ~7 months of age. N=4-5 male mice per group. Data were analyzed by one-way ANOVA with Newman-Keuls post hoc analysis. **: p<0.01, ***: p<0.001. **d** Similar expression levels of transgenic APP protein in both cortex and dorsal hippocampus of 5xFAD mouse brain with VGF germline overexpression. N=4 mice per group, 10 month old. **e** No significant difference of transgenic APP protein levels in the dorsal hippocampus of 5xFAD mouse brain with AAV-VGF overexpression. N=4 mice/per group, 10 month old. **f** Analysis of synaptic responses in the dHc of 8-9 month old 5xFAD and WT mice treated with AAV-VGF or AAV-GFP. Input/output curve expressed as fEPSP slope (mV/ms) plotted against stimulus intensity (μA) did not show differences in baseline synaptic strength between groups. N: WT (AAV-GFP) = 12 slices from 5 mice; 5xFAD (AAV-GFP) = 13 slices from 4 mice; WT (AAV-VGF) = 13 slices from 4 mice; 5xFAD (AAV-VGF) = 13 slices from 4 mice. Two-way ANOVA and Bonferroni post-hoc tests.

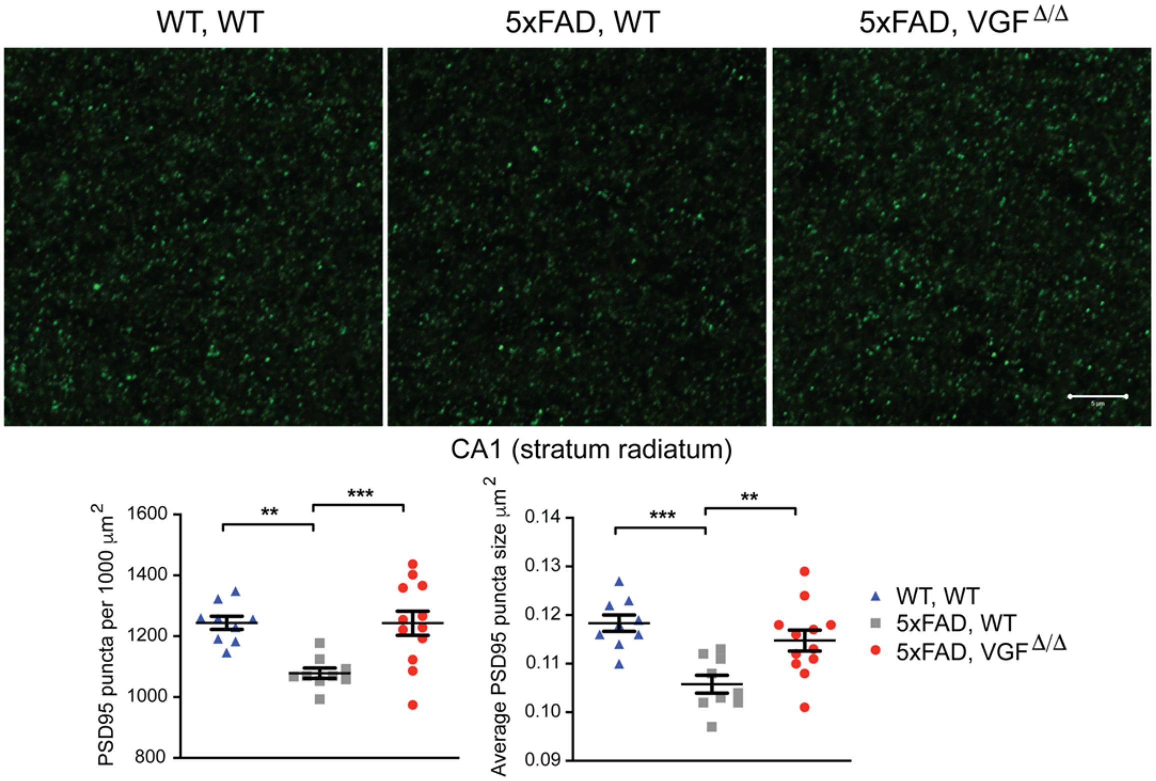

Supplementary Fig. 5.

Increased levels of PSD95 (post-synaptic density 95) in hippocampus of VGF-overexpressing 5xFAD,VGF$^{\Delta/\Delta}$ compared to 5xFAD. Levels of PSD-95 (average puncta size and puncta per 1000 μm^2) were quantified in CA1 area (stratum radiatum); n=3~4 10-month old male mice per group; 3 random fields per CA1 per brain, N=9-12; One-way ANOVA with Newman-Keuls posthoc analysis; **, $p<0.01$***, $p<0.001$; Green:PSD-95 ; Scale bar: 5μm.

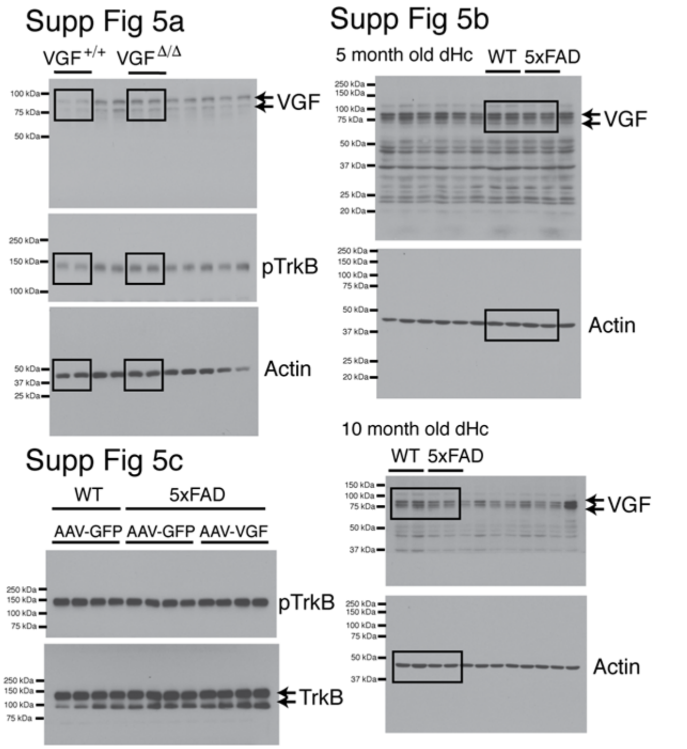

Supplementary Fig. 6.

Full length western blots of Supplementary Figure 5a-5c. Lanes shown in Figure 5a-c are highlighted by the black rectangles.

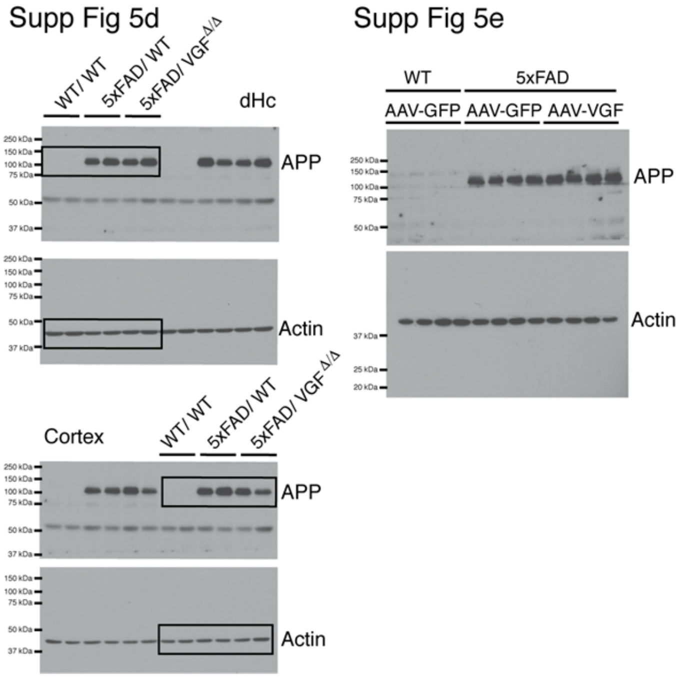

Supplementary Fig. 7.

Full length western blots of Supplementary Figure 5d-5e. Lanes shown in Figure 5 are highlighted by

the black rectangles.

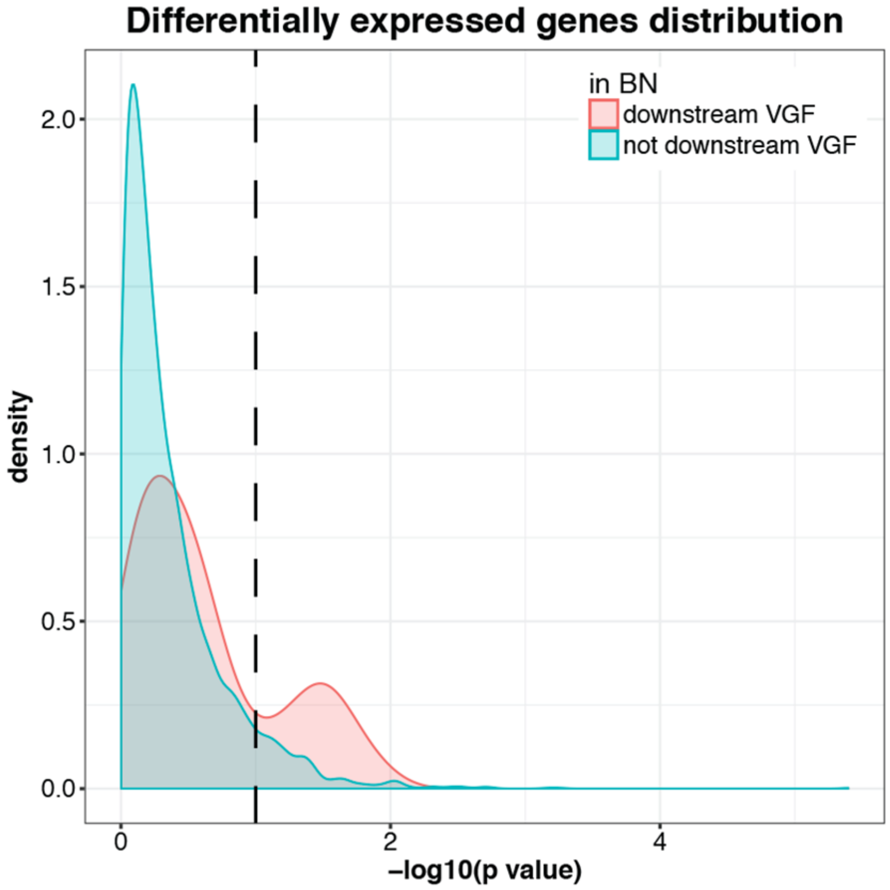

Supplementary Fig. 8.

Molecular validation VGF with germline overexpression model. Density plot of the distribution of differential expression nominal p-values for genes downstream and not downstream (causally independent of the expression levels) of VGF in the gene-only network for mouse DE genes (5xFAD, WT versus 5xFAD, VGFΔ/Δ brains): The x axis is the –log10(p-value) and the y axis the densities. The red and blue curves are for genes downstream and not downstream of VGF in the network respectively.

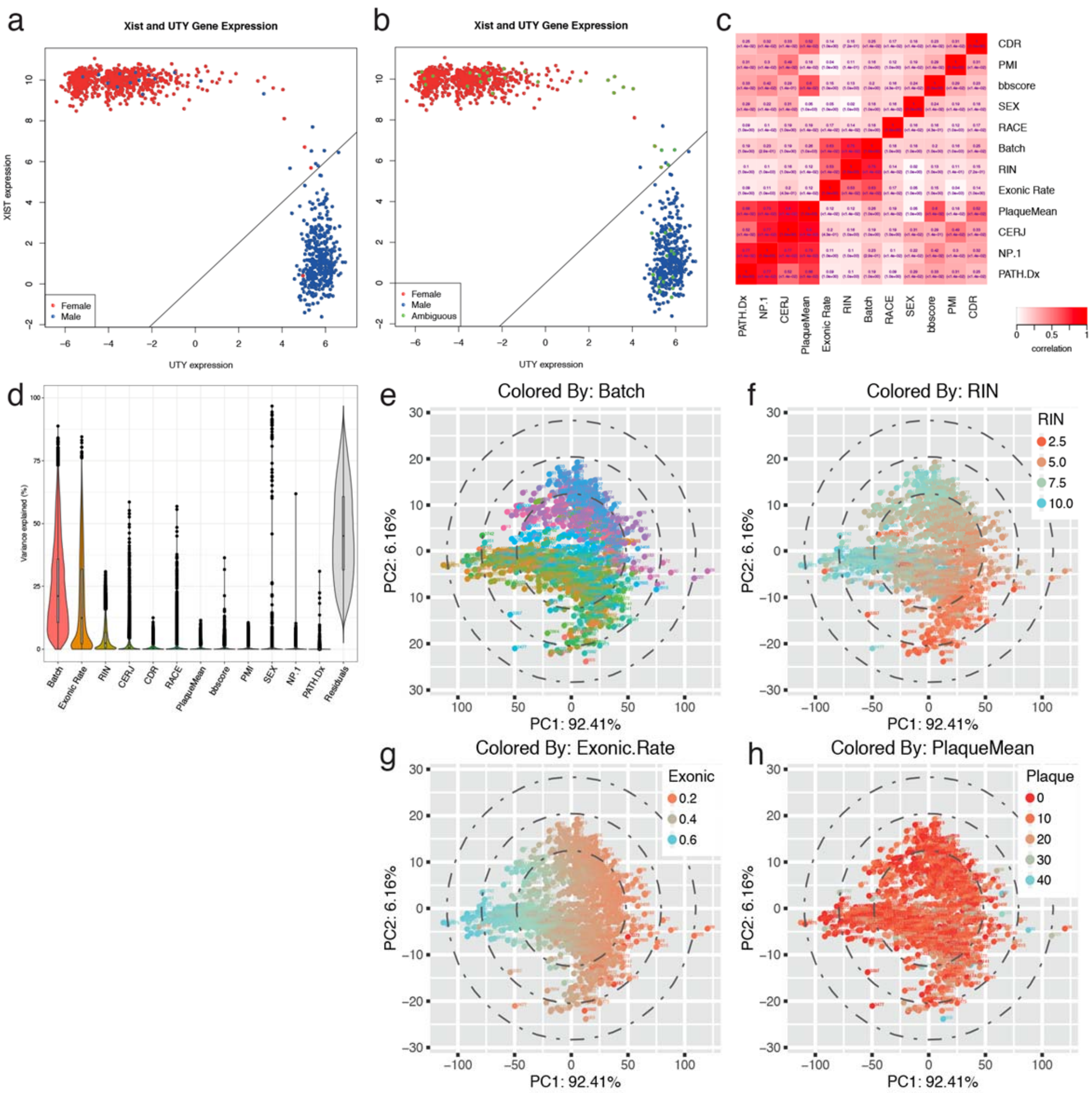

Supplementary Fig. 9.

Data quality control. a and b Imputed RNA-seq sex colored by sex clinical information: Normalized gene expression for XIST (female specific gene, y axis) and UTY (ubiquitously expressed Y-chromosome gene, male specific, x axis). **a** Obvious sex mislabeling is present in the dataset. **b** After fixing the mislabeling, ambiguous samples (removed from further analyses) are shown in green. **c** Canonical correlation heatmap of disease traits and covariates included in the model. The intensity of the red color indicates the strength of the correlation between traits and the canonical correlation (parentheses indicate Bonferroni adjusted permutation p-value) is indicated in each box. The x and y axes represent the traits and covariates: clinical dementia rating (CDR), post mortem interval (PMI), Braak score (bbscore), sex, race, batch, RNA integrity number (RIN), exonic mapping rate (exonic rate), mean neocortical plaque density

(number of plaques/mm2, PlaqueMean), CERAD neuropath Criteria (CERJ), neuropathology category (NP.1), clinical neuropathology (PATH.Dx). **d** Variance-partition violin plots of the disease traits and covariates included in the model. **e, f, g and h** Principal component analyses of important covariates: panels of this Fig. represent the same samples (one sample per point). The x axis is PC1 and explains 92.41% of the variance in the expression data. The y axis is PC2 and explains 6.16% of the variance in the expression data. The samples are colored by different QC or clinical information associated to them.

Supplementary Tables

Supplementary Table 1. Full Reference List for Approaches Shown in Introduction, Results, and Methods

Section	Approach	Full References
Introduction	Integrative biology approaches	Modeling of correlated traits vs causally related traits[5-20]; eQTL as a systematic perturbation source[6,8,9,11,21-38].
Methods	Data Description	Ribo-Zero[39]; CDR, Path Dx, CERAD neuropath CERJ, neuropath NP-1, mean neocortical plaque density, Braak score[40-45]; STAR alignement[46] ;featureCounts[47]; GATK[48]; voom and lmFit functions from limma R[49-51].
	DE analyses	limma package after the adjustment for covariates[51]; GOtest[52]; msigdb[53]; public DE sets genes[11,54-61] and proteins[62-68]; AD GWAS[69]; GWAS in TAD set [70]; locus R^2 [71,72].
	RNA Seq Processing	Main drivers of variance were explored using principal component (PC) analyses and linear mixed models (variancePartition)[73]
	QTL analyses	fastQTL package[74]; plink2[75,76]; European individuals only used to find QTLs[77]; non-European samples identified through PCA analyses using smartPCA and mapping in PC space to the 1000 Genomes Project consortium[78,79]; VCF-liftover of ROSMAP WGS from hg19 to hg38[80]; PEER surrogate (latent) variable (SVs) correction[81] ; FDR computed following Benjamini-Hochberg procedure[82].
	Co-expression analyses	coexpp R package[83,84]
	Seeding gene list construction	PEXA[85]; PPI network from CPDB[86,87].
	Bayesian Causal Network (BN)	RIMBANET[36-38,88]; Cytoscape v3.5.1[89].
	Key Driver Analyses	R package KDA[90,91]; distances function of the igraph R package [92]
	Random Forest Classifiers	data stratified by class[93]; SMOTE[94,95]; python sklearn package[96,97]; ROC curve quantification[98]; information gain score[99]; weighted z-score method[100,101].
	Polygenic risk score analyses	WGS[1]; Plink2[75,76]; I-GAP AD GWAS summary statistics[102]; PRSice2[103].
	Statistical Analyses	R v3.3.1[104]; GO annotations enrichment tested with R packages goseq[105], topGO[106] and org.Hs.eg.db[107]; MSigDB pathway enrichment tested with R packages HTSanalyzeR[108], GSEABase[109], and gage[110] ; figures generated using R packages ggplot2[111], scales[112], reshape2[113] (http://www.jstatsoft.org/v21/i12/.) and grid[114]. UpsetR plots generated with UpSetR R package[115]; heatmaps

		produced with function heatmap.2 of the R package gplots[116]; Venn diagrams were dawn using VennDiagram R package[117]; Circos (circular) plot of DE enrichments in modules plotted using NetWeaver R package[118,119]; Canonical Correlation analyses performed with the canCorPairs function of the variancePartition R package[73] and canonical correlation p-values computed with the p.perm function of the CCP R package[120] with 10,000 random sampling of the labels; large tables were read-in and written using the R package data.table[121].
	Animal models and stereotaxic surgery	Cannula implantation in the lateral ventricle [AP=−0.1, ML=±1.0 and DV: −3.0 from bregma (mm)][122]
	Immunohisto-chemical and biochemical analyses	Immunohistochemical and biochemical characterization[123-127]
	Behavioral testing and analysis	Barnes Maze test was performed using a standard apparatus[128,129]
	Field electro-physiology	Coronal brain slices containing the hippocampal formation were prepared as previously described[130]
Results	Causal network relationships	RIMBANET[6-8,11,22,23,31,33,36,38,88,131]; structure priors[6,8,11-20,29,31,36-38,88]; power boosting to infer causal relationships[6,8,11,29,31,36-38,88]; QTL perturbation to enhance causal inference among molecular traits across a broad range of diseases and data types[6-8,11,21,23,25,28,29,31,32,34,35,37,38,88,132-139].
	DE sets	Study-specific sets of DE for significantly up- and down-regulated genes[11,54-61] and proteins[62-68]
	Bayesian network (BN)	Use of BNs to capture linear and higher order correlations, nonlinear relationships, and infer causal links[8,11,14,17,22,29,36,38,88,140,141].
	Molecular Validation	Gene expression signatures induced by perturbing KDs can be compared to network predicted changes[8,11,22,28,29,31]

Supplementary Table 2.

Classification of AD. This table defines the thresholds of each disease trait for the classification of samples in disease categories [118,142] (for ROSMAP details, see https://www.synapse.org/#!Synapse:syn3191090). A full list of samples per disease trait and category can be found in Supplementary Data 1.

Dataset	classifier	controls	AD	definite controls	definite AD (dAD)
MSBB	PlaqueMean	continuous	continuous	< 6	>= 12
MSBB	CDR	< 1	>= 1	0	>= 1
MSBB	CERJ	< 2	>= 2	1	2
MSBB	Path DX	controls	non-controls	controls	dAD
MSBB	bbscore	< 3	>= 3	< 3	>= 3
MSBB	NP-1	< 2	>= 2	1	2
ROSMAP	Braaksc	< 3	>= 3	< 3	>= 3
ROSMAP	Ceradsc	>= 4	< 4	4	1
ROSMAP	Cogdx	< 4	>= 4	1	[4, 5]

Supplementary Table 3. Network Proteins and their Potential Roles in Alzheimer's Disease

Protein	Roles in AD and References
ANK2	PIK3C3-ankyrin-B-dynactin pathway promotes axonal growth and multiorganelle transport[143]
GFAP	Neuronal expression of GFAP in patients with Alzheimer pathology and identification of novel GFAP splice forms[69]
GSN	Plasma gelsolin and matrix metalloproteinase 3 are potential biomarkers for Alzheimer disease[107]
HOPX	Modulates hippocampal neurogenesis[144]
HSPB1	Modulates amyloid-beta protein precursor expression[67]
HSPB6	Neuroprotective and increases dendritic complexity[145,146]
MAOB	Monoamine oxidase-B inhibition in Alzheimer's disease[108]
PAD12	Abnormal accumulation of citrullinated proteins catalyzed by peptidylarginine deiminase in hippocampal extracts from patients with Alzheimer's disease[147,148]
PLXNB1	Semaphorin 4D-plexin-B signalling complex regulates dendritic and axonal complexity[149,150]
RPH3A	Decreased rabphilin 3A immunoreactivity in Alzheimer's disease is associated with Abeta burden[71]; involved in trafficking and release of neuronal synaptic or dense core vesicles[151,152]
SCG2	Critical for DCV biogenesis and the regulated secretion of neurotrophins, neuropeptides, and/or catecholamines[153]; required for neuronal differentiation and neural progenitor maturation[154]; reduced levels in Alzheimer's disease patient temporal cortex[91]
STXBP5L	STXBP5L (Tomosyn) involved in trafficking and release of neuronal synaptic or dense core vesicles[151,152]
SYT1	Synaptotagmins interact with APP and promote amyloid-beta generation[155]
TAGLN3	Neuronal protein 22/25 (TAGLN3) interacts with F-actin[156,157]
VGF	Interacts with amyloid precursor-like protein 1 (APLP1)[158]; critical for DCV biogenesis and the regulated secretion of neurotrophins, neuropeptides, and/or catecholamines[153]; VGF levels in CSF are reduced prospectively in patients with mild cognitive impairment, selectively in those who develop AD[159,160] and in AD[159,161-164 159,165]; VGF levels in plasma are reduced in Parkinson's disease[166], amyotrophic lateral sclerosis (ALS)[167], and major depressive disorder (MDD)[168], and are regulated by obesity and type 2 diabetes[169].

Supplementary Table 4. Network Genes and their Potential Roles in Alzheimer's Disease

Gene	Regulation	Roles in AD and Full References
BDNF	CRE/CREB[170]	Neuroprotective effects against Aβ insults[170]; BDNF plus increased adult hippocampal neurogenesis and exercise improves cognition in 5xFAD[171]; BDNF Val66Met SNP modulates neuropathology and cognitive decline in subjects with AD[172]; BDNF/TrkB signaling plays a critical role in memory and Alzheimer's disease[173]
CLU		Genome-wide association study identifies variants at CLU and PICALM associated with Alzheimer's disease[174]
CRH	CRE/CREB[170]	Neuroprotective effects against Aβ insults[171]
DUSP4	CRE/CREB[170]	DUSP4 knockout mice have spatial reference and working memory deficits[175]
DUSP6		DUSP6 is expressed in microglia and is regulated by BDNF gene ablation in PFC[176,177]; DUSP6 levels reduced in brains of Alzheimer's Disease patients[178]
FOSB	CRE/CREB[170]	DeltaFosB regulates gene expression and cognitive dysfunction in a mouse model of Alzheimer's disease[179]
GNG4		Implicated in cognitive decline during aging[180] and downregulated in aged 5xFAD mice compared to age-matched WT[181]
GRASP		GRASP (tamalin) is a scaffold protein that interacts with metabotropic glutamate receptors and regulates synaptic function[182]
MSK1 (RPS6KA5)		Mitogen- and stress-activated kinase (MSK1 or RPS6KA5) regulates BDNF signaling to CREB[183], hippocampal neurogenesis[184], synaptic plasticity[185], and cognition[186]
NPTX2 (NARP)	CRE/CREB[170]	Reduced CSF and cerebral cortical NPTX2 correlated with cognitive dysfunction in Alzheimer's Disease[187]
PTK2B (PYK2)		Pyk2 overexpression in 5xFAD Hc improves synaptic markers and behavioral performance[188]; Pyk2 mediates amyloid-β-induced synaptic dysfunction and loss[189]; Pyk2 is a novel tau tyrosine kinase[190]; in a functional screen of Alzheimer risk loci, PTK2B acts as an early marker and in vivo modulator of Tau toxicity[191]
RPH3A		Decreased rabphilin 3A immunoreactivity in Alzheimer's disease is associated with Abeta burden[71]
SCG2	CRE/CREB[170]	Critical for DCV biogenesis and the regulated secretion of neurotrophins, neuropeptides, and/or catecholamines[153]; required for neuronal differentiation and neural progenitor maturation[154]; reduced levels in Alzheimer's disease patient temporal cortex[91]
SST	CRE/CREB[170]	Somatostatin-like immunoreactivity reduced in cerebral cortex from Alzheimer's disease patients[192]
TAC1	CRE/CREB[170]	Encoding pre-protachykinin 1 peptide precursor with gene expression reduced in AD brain[193]
VGF	CRE/CREB[170]	Critical for DCV biogenesis and the regulated secretion of neurotrophins, neuropeptides, and/or catecholamines[153]; interacts with amyloid precursor-like protein 1 (APLP1)[158]

Supplementary References

1. Wang M, Beckmann ND, Roussos P, et al. The Mount Sinai cohort of large-scale genomic, transcriptomic and proteomic data in Alzheimer's disease. *Sci Data.* 2018;5:180185.
2. An JJ, Gharami K, Liao GY, et al. Distinct role of long 3' UTR BDNF mRNA in spine morphology and synaptic plasticity in hippocampal neurons. *Cell.* 2008;134(1):175-187.
3. Cajigas IJ, Tushev G, Will TJ, tom Dieck S, Fuerst N, Schuman EM. The local transcriptome in the synaptic neuropil revealed by deep sequencing and high-resolution imaging. *Neuron.* 2012;74(3):453-466.
4. Orefice LL, Waterhouse EG, Partridge JG, Lalchandani RR, Vicini S, Xu B. Distinct roles for somatically and dendritically synthesized brain-derived neurotrophic factor in morphogenesis of dendritic spines. *J Neurosci.* 2013;33(28):11618-11632.
5. Califano A, Butte AJ, Friend S, Ideker T, Schadt E. Leveraging models of cell regulation and GWAS data in integrative network-based association studies. *Nat Genet.* 2012;44(8):841-847.
6. Franzen O, Ermel R, Cohain A, et al. Cardiometabolic risk loci share downstream cis- and trans-gene regulation across tissues and diseases. *Science.* 2016;353(6301):827-830.
7. Miller CL, Pjanic M, Wang T, et al. Integrative functional genomics identifies regulatory mechanisms at coronary artery disease loci. *Nat Commun.* 2016;7:12092.
8. Peters LA, Perrigoue J, Mortha A, et al. A functional genomics predictive network model identifies regulators of inflammatory bowel disease. *Nat Genet.* 2017;49(10):1437-1449.
9. Readhead B, Haure-Mirande JV, Funk CC, et al. Multiscale Analysis of Independent Alzheimer's Cohorts Finds Disruption of Molecular, Genetic, and Clinical Networks by Human Herpesvirus. *Neuron.* 2018;99(1):64-82 e67.
10. Schadt EE. Molecular networks as sensors and drivers of common human diseases. *Nature.* 2009;461(7261):218-223.
11. Zhang B, Gaiteri C, Bodea LG, et al. Integrated systems approach identifies genetic nodes and networks in late-onset Alzheimer's disease. *Cell.* 2013;153(3):707-720.
12. Chen JC, Alvarez MJ, Talos F, et al. Identification of causal genetic drivers of human disease through systems-level analysis of regulatory networks. *Cell.* 2014;159(2):402-414.
13. Huang JK, Carlin DE, Yu MK, et al. Systematic Evaluation of Molecular Networks for Discovery of Disease Genes. *Cell Syst.* 2018;6(4):484-495 e485.
14. Meng Q, Wang K, Brunetti T, et al. The DGCR5 long noncoding RNA may regulate expression of several schizophrenia-related genes. *Sci Transl Med.* 2018;10(472).
15. Pokrovskii M, Hall JA, Ochayon DE, et al. Characterization of Transcriptional Regulatory Networks that Promote and Restrict Identities and Functions of Intestinal Innate Lymphoid Cells. *Immunity.* 2019;51(1):185-197 e186.
16. Repunte-Canonigo V, Shin W, Vendruscolo LF, et al. Identifying candidate drivers of alcohol dependence-induced excessive drinking by assembly and interrogation of brain-specific regulatory networks. *Genome Biol.* 2015;16:68.
17. Scarpa JR, Jiang P, Gao VD, et al. Cross-species systems analysis identifies gene networks differentially altered by sleep loss and depression. *Sci Adv.* 2018;4(7):eaat1294.
18. Shu L, Blencowe M, Yang X. Translating GWAS Findings to Novel Therapeutic Targets for Coronary Artery Disease. *Front Cardiovasc Med.* 2018;5:56.

19. Tomljanovic Z, Patel M, Shin W, Califano A, Teich AF. ZCCHC17 is a master regulator of synaptic gene expression in Alzheimer's disease. *Bioinformatics.* 2018;34(3):367-371.
20. Walsh LA, Alvarez MJ, Sabio EY, et al. An Integrated Systems Biology Approach Identifies TRIM25 as a Key Determinant of Breast Cancer Metastasis. *Cell Rep.* 2017;20(7):1623-1640.
21. Chang R, Karr JR, Schadt EE. Causal inference in biology networks with integrated belief propagation. *Pac Symp Biocomput.* 2015:359-370.
22. Chen Y, Zhu J, Lum PY, et al. Variations in DNA elucidate molecular networks that cause disease. *Nature.* 2008;452(7186):429-435.
23. Emilsson V, Thorleifsson G, Zhang B, et al. Genetics of gene expression and its effect on disease. *Nature.* 2008;452(7186):423-428.
24. Fromer M, Roussos P, Sieberts SK, et al. Gene expression elucidates functional impact of polygenic risk for schizophrenia. *Nat Neurosci.* 2016;19(11):1442-1453.
25. Ghazalpour A, Doss S, Zhang B, et al. Integrating genetic and network analysis to characterize genes related to mouse weight. *PLoS Genet.* 2006;2(8):e130.
26. Greenawalt DM, Dobrin R, Chudin E, et al. A survey of the genetics of stomach, liver, and adipose gene expression from a morbidly obese cohort. *Genome Res.* 2011;21(7):1008-1016.
27. Grundberg E, Small KS, Hedman AK, et al. Mapping cis- and trans-regulatory effects across multiple tissues in twins. *Nat Genet.* 2012;44(10):1084-1089.
28. Mehrabian M, Allayee H, Stockton J, et al. Integrating genotypic and expression data in a segregating mouse population to identify 5-lipoxygenase as a susceptibility gene for obesity and bone traits. *Nat Genet.* 2005;37(11):1224-1233.
29. Schadt EE, Lamb J, Yang X, et al. An integrative genomics approach to infer causal associations between gene expression and disease. *Nat Genet.* 2005;37(7):710-717.
30. Wang S, Yehya N, Schadt EE, Wang H, Drake TA, Lusis AJ. Genetic and genomic analysis of a fat mass trait with complex inheritance reveals marked sex specificity. *PLoS Genet.* 2006;2(2):e15.
31. Yang X, Deignan JL, Qi H, et al. Validation of candidate causal genes for obesity that affect shared metabolic pathways and networks. *Nat Genet.* 2009;41(4):415-423.
32. Yang X, Schadt EE, Wang S, et al. Tissue-specific expression and regulation of sexually dimorphic genes in mice. *Genome Res.* 2006;16(8):995-1004.
33. Yoo S, Takikawa S, Geraghty P, et al. Integrative analysis of DNA methylation and gene expression data identifies EPAS1 as a key regulator of COPD. *PLoS Genet.* 2015;11(1):e1004898.
34. Zhong H, Beaulaurier J, Lum PY, et al. Liver and adipose expression associated SNPs are enriched for association to type 2 diabetes. *PLoS Genet.* 2010;6(5):e1000932.
35. Zhong H, Yang X, Kaplan LM, Molony C, Schadt EE. Integrating pathway analysis and genetics of gene expression for genome-wide association studies. *Am J Hum Genet.* 2010;86(4):581-591.
36. Zhu J, Sova P, Xu Q, et al. Stitching together multiple data dimensions reveals interacting metabolomic and transcriptomic networks that modulate cell regulation. *PLoS Biol.* 2012;10(4):e1001301.
37. Zhu J, Wiener MC, Zhang C, et al. Increasing the power to detect causal associations by combining genotypic and expression data in segregating populations. *PLoS Comput Biol.* 2007;3(4):e69.

38. Zhu J, Zhang B, Smith EN, et al. Integrating large-scale functional genomic data to dissect the complexity of yeast regulatory networks. *Nat Genet.* 2008;40(7):854-861.
39. Sultan M, Amstislavskiy V, Risch T, et al. Influence of RNA extraction methods and library selection schemes on RNA-seq data. *BMC Genomics.* 2014;15:675.
40. Burke WJ, Miller JP, Rubin EH, et al. Reliability of the Washington University Clinical Dementia Rating. *Arch Neurol.* 1988;45(1):31-32.
41. Murayama S, Saito Y. Neuropathological diagnostic criteria for Alzheimer's disease. *Neuropathology.* 2004;24(3):254-260.
42. Braak H, Braak E. Neuropathological stageing of Alzheimer-related changes. *Acta Neuropathol.* 1991;82(4):239-259.
43. Hardy J, Selkoe DJ. The amyloid hypothesis of Alzheimer's disease: progress and problems on the road to therapeutics. *Science.* 2002;297(5580):353-356.
44. Hyman BT, Phelps CH, Beach TG, et al. National Institute on Aging-Alzheimer's Association guidelines for the neuropathologic assessment of Alzheimer's disease. *Alzheimers Dement.* 2012;8(1):1-13.
45. Hyman BT, Trojanowski JQ. Consensus recommendations for the postmortem diagnosis of Alzheimer disease from the National Institute on Aging and the Reagan Institute Working Group on diagnostic criteria for the neuropathological assessment of Alzheimer disease. *J Neuropathol Exp Neurol.* 1997;56(10):1095-1097.
46. Dobin A, Davis CA, Schlesinger F, et al. STAR: ultrafast universal RNA-seq aligner. *Bioinformatics.* 2013;29(1):15-21.
47. Liao Y, Smyth GK, Shi W. featureCounts: an efficient general purpose program for assigning sequence reads to genomic features. *Bioinformatics.* 2014;30(7):923-930.
48. Van der Auwera GA, Carneiro MO, Hartl C, et al. From FastQ data to high confidence variant calls: the Genome Analysis Toolkit best practices pipeline. *Curr Protoc Bioinformatics.* 2013;43:11 10 11-33.
49. Robinson MD, Oshlack A. A scaling normalization method for differential expression analysis of RNA-seq data. *Genome Biol.* 2010;11(3):R25.
50. Robinson MD, McCarthy DJ, Smyth GK. edgeR: a Bioconductor package for differential expression analysis of digital gene expression data. *Bioinformatics.* 2010;26(1):139-140.
51. Ritchie ME, Phipson B, Wu D, et al. limma powers differential expression analyses for RNA-sequencing and microarray studies. *Nucleic Acids Res.* 2015;43(7):e47.
52. *GOtest: Gene Ontology and Set Enrichment Test* [computer program]. Version 1.0.62019.
53. *msigdb: MSigDB Gene Set Collections* [computer program]. Version 0.1.42017.
54. Allen M, Wang X, Burgess JD, et al. Conserved brain myelination networks are altered in Alzheimer's and other neurodegenerative diseases. *Alzheimers Dement.* 2018;14(3):352-366.
55. Avramopoulos D, Szymanski M, Wang R, Bassett S. Gene expression reveals overlap between normal aging and Alzheimer's disease genes. *Neurobiol Aging.* 2011;32(12):2319 e2327-2334.
56. Blalock EM, Geddes JW, Chen KC, Porter NM, Markesbery WR, Landfield PW. Incipient Alzheimer's disease: microarray correlation analyses reveal major transcriptional and tumor suppressor responses. *Proc Natl Acad Sci U S A.* 2004;101(7):2173-2178.

57. Colangelo V, Schurr J, Ball MJ, Pelaez RP, Bazan NG, Lukiw WJ. Gene expression profiling of 12633 genes in Alzheimer hippocampal CA1: transcription and neurotrophic factor down-regulation and up-regulation of apoptotic and pro-inflammatory signaling. *J Neurosci Res.* 2002;70(3):462-473.
58. Liang WS, Dunckley T, Beach TG, et al. Altered neuronal gene expression in brain regions differentially affected by Alzheimer's disease: a reference data set. *Physiol Genomics.* 2008;33(2):240-256.
59. Miller JA, Woltjer RL, Goodenbour JM, Horvath S, Geschwind DH. Genes and pathways underlying regional and cell type changes in Alzheimer's disease. *Genome Med.* 2013;5(5):48.
60. Mostafavi S, Gaiteri C, Sullivan SE, et al. A molecular network of the aging human brain provides insights into the pathology and cognitive decline of Alzheimer's disease. *Nat Neurosci.* 2018;21(6):811-819.
61. Satoh J, Yamamoto Y, Asahina N, Kitano S, Kino Y. RNA-Seq data mining: downregulation of NeuroD6 serves as a possible biomarker for alzheimer's disease brains. *Dis Markers.* 2014;2014:123165.
62. Dai J, Johnson ECB, Dammer EB, et al. Effects of APOE Genotype on Brain Proteomic Network and Cell Type Changes in Alzheimer's Disease. *Front Mol Neurosci.* 2018;11:454.
63. Hondius DC, van Nierop P, Li KW, et al. Profiling the human hippocampal proteome at all pathologic stages of Alzheimer's disease. *Alzheimers Dement.* 2016;12(6):654-668.
64. Johnson ECB, Dammer EB, Duong DM, et al. Deep proteomic network analysis of Alzheimer's disease brain reveals alterations in RNA binding proteins and RNA splicing associated with disease. *Mol Neurodegener.* 2018;13(1):52.
65. Mendonca CF, Kuras M, Nogueira FCS, et al. Proteomic signatures of brain regions affected by tau pathology in early and late stages of Alzheimer's disease. *Neurobiol Dis.* 2019;130:104509.
66. Seyfried NT, Dammer EB, Swarup V, et al. A Multi-network Approach Identifies Protein-Specific Co-expression in Asymptomatic and Symptomatic Alzheimer's Disease. *Cell Syst.* 2017;4(1):60-72 e64.
67. Wingo AP, Dammer EB, Breen MS, et al. Large-scale proteomic analysis of human brain identifies proteins associated with cognitive trajectory in advanced age. *Nat Commun.* 2019;10(1):1619.
68. Zhang Q, Ma C, Gearing M, Wang PG, Chin LS, Li L. Integrated proteomics and network analysis identifies protein hubs and network alterations in Alzheimer's disease. *Acta Neuropathol Commun.* 2018;6(1):19.
69. Kunkle BW, Grenier-Boley B, Sims R, et al. Genetic meta-analysis of diagnosed Alzheimer's disease identifies new risk loci and implicates Abeta, tau, immunity and lipid processing. *Nat Genet.* 2019;51(3):414-430.
70. Wang D, Liu S, Warrell J, et al. Comprehensive functional genomic resource and integrative model for the human brain. *Science.* 2018;362(6420).
71. Machiela MJ, Chanock SJ. LDlink: a web-based application for exploring population-specific haplotype structure and linking correlated alleles of possible functional variants. *Bioinformatics.* 2015;31(21):3555-3557.

72. Machiela MJ, Chanock SJ. LDassoc: an online tool for interactively exploring genome-wide association study results and prioritizing variants for functional investigation. *Bioinformatics.* 2018;34(5):887-889.

73. Hoffman GE, Schadt EE. variancePartition: interpreting drivers of variation in complex gene expression studies. *BMC Bioinformatics.* 2016;17(1):483.

74. Ongen H, Buil A, Brown AA, Dermitzakis ET, Delaneau O. Fast and efficient QTL mapper for thousands of molecular phenotypes. *Bioinformatics.* 2016;32(10):1479-1485.

75. Chang CC, Chow CC, Tellier LC, Vattikuti S, Purcell SM, Lee JJ. Second-generation PLINK: rising to the challenge of larger and richer datasets. *Gigascience.* 2015;4:7.

76. *PLINK 1.9* [computer program].

77. Price AL, Zaitlen NA, Reich D, Patterson N. New approaches to population stratification in genome-wide association studies. *Nature reviews Genetics.* 2010;11(7):459-463.

78. Patterson N, Price AL, Reich D. Population structure and eigenanalysis. *PLoS Genet.* 2006;2(12):e190.

79. The 1000 Genomes Project Consortium. A global reference for human genetic variation. *Nature.* 2015;526(7571):68-74.

80. Gilly A. VCF-liftover. 2016; https://github.com/wtsi-team144/VCF-liftover.

81. Stegle O, Parts L, Durbin R, Winn J. A Bayesian framework to account for complex non-genetic factors in gene expression levels greatly increases power in eQTL studies. *PLoS Comput Biol.* 2010;6(5):e1000770.

82. Benjamini Y, Hochberg, Y. Controlling the False Discovery Rate: A Practical and Powerful Approach to Multiple Testing. . *Journal of the Royal Statistical Society.* 1995;Series B (Methodological). .

83. Langfelder P, Horvath S. WGCNA: an R package for weighted correlation network analysis. *BMC Bioinformatics.* 2008;9:559.

84. *coexpp* [computer program]. 2011.

85. Tu Z, Argmann C, Wong KK, et al. Integrating siRNA and protein-protein interaction data to identify an expanded insulin signaling network. *Genome Res.* 2009;19(6):1057-1067.

86. Kanehisa M, Goto S. KEGG: kyoto encyclopedia of genes and genomes. *Nucleic Acids Res.* 2000;28(1):27-30.

87. Kamburov A, Stelzl U, Lehrach H, Herwig R. The ConsensusPathDB interaction database: 2013 update. *Nucleic Acids Res.* 2013;41(Database issue):D793-800.

88. Zhu J, Lum PY, Lamb J, et al. An integrative genomics approach to the reconstruction of gene networks in segregating populations. *Cytogenet Genome Res.* 2004;105(2-4):363-374.

89. Shannon P, Markiel A, Ozier O, et al. Cytoscape: a software environment for integrated models of biomolecular interaction networks. *Genome Res.* 2003;13(11):2498-2504.

90. Zhang B, Zhu J. Identification of Key Causal Regulators in Gene Networks. *Proceedings of the World Congress on Engineering & Computer Science.* 2013;2.

91. Lassmann H, Weiler R, Fischer P, et al. Synaptic pathology in Alzheimer's disease: immunological data for markers of synaptic and large dense-core vesicles. *Neuroscience.* 1992;46(1):1-8.

92. Csardi G, Nepusz T. The igraph software package for complex network research. *InterJournal.* 2006;Complex Systems:1695.

93. James G, Witten D, Hastie T, Tibshirani R. An Introduction to Statistical Learning with Applications in R Introduction. *Springer Texts Stat.* 2013;103:1-14.
94. Chawla NV, Bowyer KW, Hall LO, Kegelmeyer WP. SMOTE: Synthetic minority over-sampling technique. *J Artif Intell Res.* 2002;16:321-357.
95. Lemaitre G, Nogueira F, Aridas CK. Imbalanced-learn: A Python Toolbox to Tackle the Curse of Imbalanced Datasets in Machine Learning. *J Mach Learn Res.* 2017;18.
96. Breiman L. Random forests. *Mach Learn.* 2001;45(1):5-32.
97. Pedregosa F, Varoquaux G, Gramfort A, et al. Scikit-learn: Machine Learning in Python. *J Mach Learn Res.* 2011;12:2825-2830.
98. Bradley AP. The use of the area under the roc curve in the evaluation of machine learning algorithms. *Pattern Recogn.* 1997;30(7):1145-1159.
99. Kullback S, Leibler RA. On Information and Sufficiency. *Ann Math Stat.* 1951;22(1):79-86.
100. Stouffer SA. *The American soldier.* Princeton,: Princeton University Press; 1949.
101. T. L. On the combination of independent tests. *Magyar Tud Akad Mat Kutato Int Kozl.* 1958;3:171.
102. Lambert JC, Ibrahim-Verbaas CA, Harold D, et al. Meta-analysis of 74,046 individuals identifies 11 new susceptibility loci for Alzheimer's disease. *Nat Genet.* 2013;45(12):1452-1458.
103. Euesden J, Lewis CM, O'Reilly PF. PRSice: Polygenic Risk Score software. *Bioinformatics.* 2015;31(9):1466-1468.
104. *R: A language and environment for statistical computing* [computer program]. Version 3.3.1: R Foundation for Statistical Computing; 2016.
105. Young MD, Wakefield MJ, Smyth GK, Oshlack A. Gene ontology analysis for RNA-seq: accounting for selection bias. *Genome Biol.* 2010;11(2):R14.
106. *topGO: topGO: Enrichment analysis for Gene Ontology.* [computer program]. Version R package version 2.18.0.2010.
107. *org.Hs.eg.db: Genome wide annotation for Human.* [computer program]. Version R package version 3.2.3.
108. Wang X, Terfve C, Rose JC, Markowetz F. HTSanalyzeR: an R/Bioconductor package for integrated network analysis of high-throughput screens. *Bioinformatics.* 2011;27(6):879-880.
109. *GSEABase: Gene set enrichment data structures and methods.* [computer program]. Version R package version 1.32.0.
110. Luo W, Friedman MS, Shedden K, Hankenson KD, Woolf PJ. GAGE: generally applicable gene set enrichment for pathway analysis. *BMC Bioinformatics.* 2009;10:161.
111. Wickham H. *ggplot2: Elegant Graphics for Data Analysis.* New York: Springer-Verlag 2009.
112. *scales: Scale functions for graphics.* [computer program]. Version R package version 0.2.3. 2012.
113. Wickham H. Reshaping Data with the reshape Package. *Journal of Statistical Software.* 2007;21(12):1-20.
114. Murrell P. *R Graphics.* Boca Raton, Florida: Chapman and Hall/CRC; 2005.
115. Conway JR, Lex A, Gehlenborg N. UpSetR: An R Package for the Visualization of Intersecting Sets and their Properties. *Bioinformatics.* 2017.

116. *gplots: Various R Programming Tools for Plotting Data* [computer program]. Version 3.0.12016.
117. *VennDiagram: Generate High-Resolution Venn and Euler Plots* [computer program]. Version 1.6.172016.
118. Wang M, Roussos P, McKenzie A, et al. Integrative network analysis of nineteen brain regions identifies molecular signatures and networks underlying selective regional vulnerability to Alzheimer's disease. *Genome Med.* 2016;8(1):104.
119. *NetWeaver: Graphic Presentation of Complex Genomic and Network Data Analysis* [computer program]. 2016.
120. *CCP: Significance Tests for Canonical Correlation Analysis (CCA)* [computer program]. Version R package version 1.12012.
121. Dowle M, Srinivasan A. data.table: Extension of `data.frame`. 2019.
122. Bartolomucci A, La Corte G, Possenti R, et al. TLQP-21, a VGF-derived peptide, increases energy expenditure and prevents the early phase of diet-induced obesity. *Proc Natl Acad Sci U S A.* 2006;103(39):14584-14589.
123. Knight EM, Kim SH, Kottwitz JC, et al. Effective anti-Alzheimer Abeta therapy involves depletion of specific Abeta oligomer subtypes. *Neurol Neuroimmunol Neuroinflamm.* 2016;3(3):e237.
124. Knight EM, Ruiz HH, Kim SH, et al. Unexpected partial correction of metabolic and behavioral phenotypes of Alzheimer's APP/PSEN1 mice by gene targeting of diabetes/Alzheimer's-related Sorcs1. *Acta Neuropathol Commun.* 2016;4:16.
125. Lambert MP, Velasco PT, Chang L, et al. Monoclonal antibodies that target pathological assemblies of Abeta. *J Neurochem.* 2007;100(1):23-35.
126. Tomic JL, Pensalfini A, Head E, Glabe CG. Soluble fibrillar oligomer levels are elevated in Alzheimer's disease brain and correlate with cognitive dysfunction. *Neurobiol Dis.* 2009;35(3):352-358.
127. Haure-Mirande JV, Audrain M, Fanutza T, et al. Deficiency of TYROBP, an adapter protein for TREM2 and CR3 receptors, is neuroprotective in a mouse model of early Alzheimer's pathology. *Acta Neuropathol.* 2017;134(5):769-788.
128. Barnes CA. Memory deficits associated with senescence: a neurophysiological and behavioral study in the rat. *J Comp Physiol Psychol.* 1979;93(1):74-104.
129. Sunyer B PS, Höger H, Luber G Barnes maze, a useful task to assess spatial reference memory in the mice. *Protoc Exch.* 2007.
130. Fakira AK, Portugal GS, Carusillo B, Melyan Z, Moron JA. Increased small conductance calcium-activated potassium type 2 channel-mediated negative feedback on N-methyl-D-aspartate receptors impairs synaptic plasticity following context-dependent sensitization to morphine. *Biol Psychiatry.* 2014;75(2):105-114.
131. Narayanan M, Huynh JL, Wang K, et al. Common dysregulation network in the human prefrontal cortex underlies two neurodegenerative diseases. *Mol Syst Biol.* 2014;10:743.
132. Schadt EE, Monks SA, Drake TA, et al. Genetics of gene expression surveyed in maize, mouse and man. *Nature.* 2003;422(6929):297-302.
133. Doss S, Schadt EE, Drake TA, Lusis AJ. Cis-acting expression quantitative trait loci in mice. *Genome Res.* 2005;15(5):681-691.
134. Dobrin R, Zhu J, Molony C, et al. Multi-tissue coexpression networks reveal unexpected subnetworks associated with disease. *Genome Biol.* 2009;10(5):R55.

135. Zhang W, Zhu J, Schadt EE, Liu JS. A Bayesian partition method for detecting pleiotropic and epistatic eQTL modules. *PLoS Comput Biol.* 2010;6(1):e1000642.
136. Millstein J, Winrow CJ, Kasarskis A, et al. Identification of causal genes, networks, and transcriptional regulators of REM sleep and wake. *Sleep.* 2011;34(11):1469-1477.
137. Schadt EE, Woo S, Hao K. Bayesian method to predict individual SNP genotypes from gene expression data. *Nat Genet.* 2012;44(5):603-608.
138. Tu Z, Keller MP, Zhang C, et al. Integrative analysis of a cross-loci regulation network identifies App as a gene regulating insulin secretion from pancreatic islets. *PLoS Genet.* 2012;8(12):e1003107.
139. Roussos P, Mitchell AC, Voloudakis G, et al. A role for noncoding variation in schizophrenia. *Cell Rep.* 2014;9(4):1417-1429.
140. Petyuk VA, Chang R, Ramirez-Restrepo M, et al. The human brainome: network analysis identifies HSPA2 as a novel Alzheimer’s disease target. *Brain.* 2018;141(9):2721-2739.
141. Carcamo-Orive I, Hoffman GE, Cundiff P, et al. Analysis of Transcriptional Variability in a Large Human iPSC Library Reveals Genetic and Non-genetic Determinants of Heterogeneity. *Cell Stem Cell.* 2017;20(4):518-532 e519.
142. Haroutunian V, Katsel P, Schmeidler J. Transcriptional vulnerability of brain regions in Alzheimer's disease and dementia. *Neurobiol Aging.* 2009;30(4):561-573.
143. Lorenzo DN, Badea A, Davis J, et al. A PIK3C3-ankyrin-B-dynactin pathway promotes axonal growth and multiorganelle transport. *J Cell Biol.* 2014;207(6):735-752.
144. Li D, Takeda N, Jain R, et al. Hopx distinguishes hippocampal from lateral ventricle neural stem cells. *Stem Cell Res.* 2015;15(3):522-529.
145. Bartelt-Kirbach B, Moron M, Glomb M, Beck CM, Weller MP, Golenhofen N. HspB5/alphaB-crystallin increases dendritic complexity and protects the dendritic arbor during heat shock in cultured rat hippocampal neurons. *Cell Mol Life Sci.* 2016;73(19):3761-3775.
146. Qi AQ, Zhang YH, Qi QD, Liu YH, Zhu JL. Overexpressed HspB6 Underlines a Novel Inhibitory Role in Kainic Acid-Induced Epileptic Seizure in Rats by Activating the cAMP-PKA Pathway. *Cell Mol Neurobiol.* 2019;39(1):111-122.
147. Ishigami A, Ohsawa T, Hiratsuka M, et al. Abnormal accumulation of citrullinated proteins catalyzed by peptidylarginine deiminase in hippocampal extracts from patients with Alzheimer's disease. *J Neurosci Res.* 2005;80(1):120-128.
148. Tu R, Grover HM, Kotra LP. Peptidyl Arginine Deiminases and Neurodegenerative Diseases. *Curr Med Chem.* 2016;23(2):104-114.
149. Vodrazka P, Korostylev A, Hirschberg A, et al. The semaphorin 4D-plexin-B signalling complex regulates dendritic and axonal complexity in developing neurons via diverse pathways. *Eur J Neurosci.* 2009;30(7):1193-1208.
150. Swiercz JM, Kuner R, Offermanns S. Plexin-B1/RhoGEF-mediated RhoA activation involves the receptor tyrosine kinase ErbB-2. *J Cell Biol.* 2004;165(6):869-880.
151. Geerts CJ, Mancini R, Chen N, et al. Tomosyn associates with secretory vesicles in neurons through its N- and C-terminal domains. *PLoS One.* 2017;12(7):e0180912.
152. Burns ME, Sasaki T, Takai Y, Augustine GJ. Rabphilin-3A: a multifunctional regulator of synaptic vesicle traffic. *J Gen Physiol.* 1998;111(2):243-255.

153. Bartolomucci A, Possenti R, Mahata SK, Fischer-Colbrie R, Loh YP, Salton SR. The extended granin family: structure, function, and biomedical implications. *Endocr Rev.* 2011;32(6):755-797.

154. Kim HJ, Denli AM, Wright R, et al. REST Regulates Non-Cell-Autonomous Neuronal Differentiation and Maturation of Neural Progenitor Cells via Secretogranin II. *J Neurosci.* 2015;35(44):14872-14884.

155. Gautam V, D'Avanzo C, Berezovska O, Tanzi RE, Kovacs DM. Synaptotagmins interact with APP and promote Abeta generation. *Mol Neurodegener.* 2015;10:31.

156. Mori K, Muto Y, Kokuzawa J, et al. Neuronal protein NP25 interacts with F-actin. *Neurosci Res.* 2004;48(4):439-446.

157. Depaz IM, Wilce PA. The novel cytoskeleton-associated protein Neuronal protein 22: elevated expression in the developing rat brain. *Brain Res.* 2006;1081(1):59-64.

158. Bai Y, Markham K, Chen F, et al. The in vivo brain interactome of the amyloid precursor protein. *Mol Cell Proteomics.* 2008;7(1):15-34.

159. Jahn H, Wittke S, Zurbig P, et al. Peptide fingerprinting of Alzheimer's disease in cerebrospinal fluid: identification and prospective evaluation of new synaptic biomarkers. *PLoS One.* 2011;6(10):e26540.

160. Llano DA, Bundela S, Mudar RA, Devanarayan V, Alzheimer's Disease Neuroimaging I. A multivariate predictive modeling approach reveals a novel CSF peptide signature for both Alzheimer's Disease state classification and for predicting future disease progression. *PLoS One.* 2017;12(8):e0182098.

161. Carrette O, Demalte I, Scherl A, et al. A panel of cerebrospinal fluid potential biomarkers for the diagnosis of Alzheimer's disease. *Proteomics.* 2003;3(8):1486-1494.

162. Hendrickson RC, Lee AY, Song Q, et al. High Resolution Discovery Proteomics Reveals Candidate Disease Progression Markers of Alzheimer's Disease in Human Cerebrospinal Fluid. *PLoS One.* 2015;10(8):e0135365.

163. Holtta M, Minthon L, Hansson O, et al. An integrated workflow for multiplex CSF proteomics and peptidomics-identification of candidate cerebrospinal fluid biomarkers of Alzheimer's disease. *J Proteome Res.* 2015;14(2):654-663.

164. Spellman DS, Wildsmith KR, Honigberg LA, et al. Development and evaluation of a multiplexed mass spectrometry based assay for measuring candidate peptide biomarkers in Alzheimer's Disease Neuroimaging Initiative (ADNI) CSF. *Proteomics Clin Appl.* 2015;9(7-8):715-731.

165. Duits FH, Brinkmalm G, Teunissen CE, et al. Synaptic proteins in CSF as potential novel biomarkers for prognosis in prodromal Alzheimer's disease. *Alzheimers Res Ther.* 2018;10(1):5.

166. Cocco C, Corda G, Lisci C, et al. VGF peptides as novel biomarkers in Parkinson's disease. *Cell Tissue Res.* 2020;379(1):93-107.

167. Brancia C, Noli B, Boido M, et al. TLQP Peptides in Amyotrophic Lateral Sclerosis: Possible Blood Biomarkers with a Neuroprotective Role. *Neuroscience.* 2018;380:152-163.

168. Jiang H, Chen S, Lu N, et al. Reduced serum VGF levels were reversed by antidepressant treatment in depressed patients. *World J Biol Psychiatry.* 2017;18(8):586-591.

169. D'Amato F, Noli B, Angioni L, et al. VGF Peptide Profiles in Type 2 Diabetic Patients' Plasma and in Obese Mice. *PLoS One.* 2015;10(11):e0142333.

170. Benito E, Valor LM, Jimenez-Minchan M, Huber W, Barco A. cAMP response element-binding protein is a primary hub of activity-driven neuronal gene expression. *J Neurosci.* 2011;31(50):18237-18250.
171. Choi SH, Bylykbashi E, Chatila ZK, et al. Combined adult neurogenesis and BDNF mimic exercise effects on cognition in an Alzheimer's mouse model. *Science.* 2018;361(6406).
172. Franzmeier N, Ren J, Damm A, et al. The BDNFVal66Met SNP modulates the association between beta-amyloid and hippocampal disconnection in Alzheimer's disease. *Mol Psychiatry.* 2019.
173. Zhang F, Kang Z, Li W, Xiao Z, Zhou X. Roles of brain-derived neurotrophic factor/tropomyosin-related kinase B (BDNF/TrkB) signalling in Alzheimer's disease. *J Clin Neurosci.* 2012;19(7):946-949.
174. Harold D, Abraham R, Hollingworth P, et al. Genome-wide association study identifies variants at CLU and PICALM associated with Alzheimer's disease. *Nat Genet.* 2009;41(10):1088-1093.
175. Abdul Rahman NZ, Greenwood SM, Brett RR, et al. Mitogen-Activated Protein Kinase Phosphatase-2 Deletion Impairs Synaptic Plasticity and Hippocampal-Dependent Memory. *J Neurosci.* 2016;36(8):2348-2354.
176. Ham JE, Oh EK, Kim DH, Choi SH. Differential expression profiles and roles of inducible DUSPs and ERK1/2-specific constitutive DUSP6 and DUSP7 in microglia. *Biochem Biophys Res Commun.* 2015;467(2):254-260.
177. Glorioso C, Sabatini M, Unger T, et al. Specificity and timing of neocortical transcriptome changes in response to BDNF gene ablation during embryogenesis or adulthood. *Mol Psychiatry.* 2006;11(7):633-648.
178. Banzhaf-Strathmann J, Benito E, May S, et al. MicroRNA-125b induces tau hyperphosphorylation and cognitive deficits in Alzheimer's disease. *EMBO J.* 2014;33(15):1667-1680.
179. Corbett BF, You JC, Zhang X, et al. DeltaFosB Regulates Gene Expression and Cognitive Dysfunction in a Mouse Model of Alzheimer's Disease. *Cell Rep.* 2017;20(2):344-355.
180. Bonham LW, Evans DS, Liu Y, Cummings SR, Yaffe K, Yokoyama JS. Neurotransmitter Pathway Genes in Cognitive Decline During Aging: Evidence for GNG4 and KCNQ2 Genes. *Am J Alzheimers Dis Other Demen.* 2018;33(3):153-165.
181. Bouter Y, Kacprowski T, Weissmann R, et al. Deciphering the molecular profile of plaques, memory decline and neuron loss in two mouse models for Alzheimer's disease by deep sequencing. *Front Aging Neurosci.* 2014;6:75.
182. Kitano J, Kimura K, Yamazaki Y, et al. Tamalin, a PDZ domain-containing protein, links a protein complex formation of group 1 metabotropic glutamate receptors and the guanine nucleotide exchange factor cytohesins. *J Neurosci.* 2002;22(4):1280-1289.
183. Daumas S, Hunter CJ, Mistry RB, et al. The Kinase Function of MSK1 Regulates BDNF Signaling to CREB and Basal Synaptic Transmission, But Is Not Required for Hippocampal Long-Term Potentiation or Spatial Memory. *eNeuro.* 2017;4(1).
184. Choi YS, Karelina K, Alzate-Correa D, et al. Mitogen- and stress-activated kinases regulate progenitor cell proliferation and neuron development in the adult dentate gyrus. *J Neurochem.* 2012;123(5):676-688.

185. Correa SA, Hunter CJ, Palygin O, et al. MSK1 regulates homeostatic and experience-dependent synaptic plasticity. *J Neurosci.* 2012;32(38):13039-13051.
186. Karelina K, Hansen KF, Choi YS, DeVries AC, Arthur JS, Obrietan K. MSK1 regulates environmental enrichment-induced hippocampal plasticity and cognitive enhancement. *Learn Mem.* 2012;19(11):550-560.
187. Xiao MF, Xu D, Craig MT, et al. NPTX2 and cognitive dysfunction in Alzheimer's Disease. *Elife.* 2017;6.
188. Giralt A, de Pins B, Cifuentes-Diaz C, et al. PTK2B/Pyk2 overexpression improves a mouse model of Alzheimer's disease. *Exp Neurol.* 2018;307:62-73.
189. Salazar SV, Cox TO, Lee S, et al. Alzheimer's Disease Risk Factor Pyk2 Mediates Amyloid-beta-Induced Synaptic Dysfunction and Loss. *J Neurosci.* 2019;39(4):758-772.
190. Li C, Gotz J. Pyk2 is a Novel Tau Tyrosine Kinase that is Regulated by the Tyrosine Kinase Fyn. *J Alzheimers Dis.* 2018;64(1):205-221.
191. Dourlen P, Fernandez-Gomez FJ, Dupont C, et al. Functional screening of Alzheimer risk loci identifies PTK2B as an in vivo modulator and early marker of Tau pathology. *Mol Psychiatry.* 2017;22(6):874-883.
192. Davies P, Katzman R, Terry RD. Reduced somatostatin-like immunoreactivity in cerebral cortex from cases of Alzheimer disease and Alzheimer senile dementa. *Nature.* 1980;288(5788):279-280.
193. Magistri M, Velmeshev D, Makhmutova M, Faghihi MA. Transcriptomics Profiling of Alzheimer's Disease Reveal Neurovascular Defects, Altered Amyloid-beta Homeostasis, and Deregulated Expression of Long Noncoding RNAs. *J Alzheimers Dis.* 2015;48(3):647-665.
