# Supplementary material for: Multiscale causal networks identify VGF as a key regulator of Alzheimer’s disease
Source: Nat Commun. 2020 Aug 7;11:3942. doi: 10.1038/s41467-020-17405-z (PMC7414858; doi:10.1038/s41467-020-17405-z)
Supplement: Supplementary file 2 — Reporting Summary [file 41467_2020_17405_MOESM2_ESM.pdf]

nature research

Corresponding author(s): Eric Schadt

Last updated by author(s): 03/02/2020

Reporting Summary

Nature Research wishes to improve the reproducibility of the work that we publish. This form provides structure for consistency and transparency in reporting. For further information on Nature Research policies, see Authors & Referees and the Editorial Policy Checklist .

Statistics

For all statistical analyses, confirm that the following items are present in the figure legend, table legend, main text, or Methods section.

n/a	Confirmed	
☐	☒	The exact sample size (n) for each experimental group/condition, given as a discrete number and unit of measurement
☐	☒	A statement on whether measurements were taken from distinct samples or whether the same sample was measured repeatedly
☐	☒	The statistical test(s) used AND whether they are one- or two-sided<br>*Only common tests should be described solely by name; describe more complex techniques in the Methods section.*
☐	☒	A description of all covariates tested
☐	☒	A description of any assumptions or corrections, such as tests of normality and adjustment for multiple comparisons
☐	☒	A full description of the statistical parameters including central tendency (e.g. means) or other basic estimates (e.g. regression coefficient) AND variation (e.g. standard deviation) or associated estimates of uncertainty (e.g. confidence intervals)
☐	☒	For null hypothesis testing, the test statistic (e.g. F, t, r) with confidence intervals, effect sizes, degrees of freedom and P value noted<br>*Give P values as exact values whenever suitable.*
☐	☒	For Bayesian analysis, information on the choice of priors and Markov chain Monte Carlo settings
☐	☒	For hierarchical and complex designs, identification of the appropriate level for tests and full reporting of outcomes
☐	☒	Estimates of effect sizes (e.g. Cohen's d, Pearson's r), indicating how they were calculated

Our web collection on statistics for biologists contains articles on many of the points above.

Software and code

Policy information about availability of computer code

Data collection	No software was used
Data analysis	No commercial code was used. All other codes used are described in the methods and online methods sections. They include R, GATK, FeatureCounts, STAR, WGCNA, RIMBANET, MaxQuant, Limma, VariancePartition, fastQTL, smartPCA, PEER, PEXA, KDA, topGO, goseq, org.Hs.eg.db, HTSanalyzeR, GSEABase, gage, ggplot2, scales, reshape2, UpsetR, VennDiagram, Netweaver, Plink, Plink2, PRSice, and cytoscape.

For manuscripts utilizing custom algorithms or software that are central to the research but not yet described in published literature, software must be made available to editors/reviewers. We strongly encourage code deposition in a community repository (e.g. GitHub). See the Nature Research guidelines for submitting code & software for further information.

Data

Policy information about availability of data

All manuscripts must include a data availability statement. This statement should provide the following information, where applicable:

- Accession codes, unique identifiers, or web links for publicly available datasets
- A list of figures that have associated raw data
- A description of any restrictions on data availability

All raw data is available at synapse.org: https://www.synapse.org/#!Synapse:syn2580853/wiki/409853

Field-specific reporting

Please select the one below that is the best fit for your research. If you are not sure, read the appropriate sections before making your selection.

Life sciences study design

All studies must disclose on these points even when the disclosure is negative.

Sample size	Based on previous work for all the methods used and power calculations (G*Power software package and PROPER R package), we determined that the sample size at hand would provide adequate power to identify novel key regulators.
Data exclusions	Data was excluded if mislabelled or not achieving minimum quality requirement.
Replication	Finding was successfully replicated in 2 other brain regions and an independent prefrontal cortex dataset as well as validated in animal model and validation was replicated with 3 different perturbations.
Randomization	Sample data was generated in large batches comprising all traits of interest, and subsequently corrected for covariates.
Blinding	Blinding was not relevant to the study, as we were looking to identify differences between the groups post-mortem and were not trying to assess effects of treatment on the disease.

Reporting for specific materials, systems and methods

We require information from authors about some types of materials, experimental systems and methods used in many studies. Here, indicate whether each material, system or method listed is relevant to your study. If you are not sure if a list item applies to your research, read the appropriate section before selecting a response.

Materials & experimental systems

n/a	Involved in the study	
☐	☒	Antibodies
☒	☐	Eukaryotic cell lines
☒	☐	Palaeontology
☐	☒	Animals and other organisms
☐	☒	Human research participants
☒	☐	Clinical data

Methods

n/a	Involved in the study	
☒	☐	ChIP-seq
☒	☐	Flow cytometry
☒	☐	MRI-based neuroimaging

Antibodies

Antibodies used	rabbit pAb A11 (anti-prefibrillar oligomers, 0.5 µg/ml, courtesy of Dr. Glabe), rabbit pAb OC (anti-fibrillar oligomers and fibrils; 0.25 µg/ml, courtesy of Dr. Glabe), mouse mAb Nu-4(anti-oligomers; 1 µg/ml, courtesy of Dr. Klein), mouse 6E10 antibody (1:1000; Covance, Princeton, NJ, cat# 9320-500, lot# D14FF01323), rabbit anti-Iba1 (1:500; Wako, Richmond, VA, cat# 019-19741, lot# WDK2121), anti-mouse Alexa Fluor 488 (1:500; Invitrogen, Carlsbad, CA, cat# A21121, lot# 1704461), anti-rabbit Alexa Fluor 594 (1:500; Invitrogen, Carlsbad, CA, cat# A11012, lot# 1745478), anti-VGF C-terminal (1:1000; rabbit polyclonal, Salton lab), anti-actin (1:1000; MilliporeSigma, cat# MAB1501, lot# 2908073) antibodies, secondary horseradish peroxidase-labeled anti-rabbit or donkey anti-mouse antibody (1/6000; GE Healthcare, anti-rabbit: cat# NA934V, lot# 9636020/9832916; anti-mouse: cat# NA931V, lot# 9648884)
Validation	rabbit pAb A11 (characterized by Tomic et al, 2009 for WB and dot blot assay), rabbit pAb OC (characterized by Tomic et al, 2009 for WB and dot blot assay), mouse mAb Nu-4(characterized by Lambert et al. and others for WB, IHC and dot blot), mouse 6E10 antibody (validated for Elisa, WB, IHC, IP and reactive to amino acid residue 1-16 of beta amyloid), rabbit anti-Iba1 (validated for IHC in human, mouse and rat), anti-VGF C-terminal (characterized by Lin et al, 2015)

Animals and other organisms

Policy information about studies involving animals; ARRIVE guidelines recommended for reporting animal research

Laboratory animals	Mouse, 5xFAD in B6/SJL background crossed with mixed background N3F1 Vgf flox/flox (>93.7% C57BL/6J background) and respective control, both males and females. Mouse, 5xFAD in B6/SJL background and controls, both males and females, injected with AAV at 2-3 month of age, analyzed at both 7,8 and 10 month old. Mouse, 5xFAD in B6/SJL background and controls, both males and females, injected with TLQP-62 (2.5 mg/ml) dissolved in aCSF or aCSF alone was delivered icv by microosmotic pump for 28 days and analyzed at 4.5 months of age.
Wild animals	*Provide details on animals observed in or captured in the field; report species, sex and age where possible. Describe how animals were caught and transported and what happened to captive animals after the study (if killed, explain why and describe method; if released, say where and when) OR state that the study did not involve wild animals.*
Field-collected samples	*For laboratory work with field-collected samples, describe all relevant parameters such as housing, maintenance, temperature, photoperiod and end-of-experiment protocol OR state that the study did not involve samples collected from the field.*

Ethics oversight	All mouse studies were conducted in accordance with the U.S. National Institutes of Health Guidelines for the Care and Use of Experimental Animals, using protocols approved by the Institutional Animal Care and Use Committee of the Icahn School of Medicine at Mount Sinai

Note that full information on the approval of the study protocol must also be provided in the manuscript.

Human research participants

Policy information about studies involving human research participants

Population characteristics	MSBB data: 315 individuals across the spectrum of Alzheimer's disease. Mean age is 84.9 years old, with 204 females and 111 males. Disease category is described in supplementary table 1, with individuals going from control to the full range of neuropathology. ROSMAP (The religious order study and memory aging project) data: Across the spectrum of Alzheimer's disease, 724 individuals with RNA-seq and 1200 individuals with whole genome sequencing.
Recruitment	*Describe how participants were recruited. Outline any potential self-selection bias or other biases that may be present and how these are likely to impact results.*
Ethics oversight	All human subject research was carried out in accordance with the policies and procedures of the Icahn School of Medicine at Mount Sinai and its Institutional Review Board

Note that full information on the approval of the study protocol must also be provided in the manuscript.
