# Supplementary material for: Multiscale causal networks identify VGF as a key regulator of Alzheimer’s disease
Source: Nat Commun. 2020 Aug 7;11:3942. doi: 10.1038/s41467-020-17405-z (PMC7414858; doi:10.1038/s41467-020-17405-z)
Supplement: Supplementary file 3 — Description of Additional Supplementary Files [file 41467_2020_17405_MOESM3_ESM.pdf]

Description of Additional Supplementary Data

Supplementary Data 1. Supplementary Data 1 Sample Breakdown.xlsx

Description of the AMP-AD MSBB population and samples measured.
The first sheet contains descriptions and hyperlinks to all sheets in the Table. Sheet 2: all_brain_regions: Description of the entire AMP-AD MSBB population and samples measured. Samples are broken down by AD traits, and the following description of groups are present: Sex, mean age (std), APOE genotype, mean post mortem interval (std), number of samples with proteomics, number of samples with WES, number of samples with RNA-seq per brain region, mean RIN per brain region (std) and mean exonic mapping rate per brain region (std). Sheet 3: BM10 only: Description of the BM10 (discovery) dataset. Samples are broken down by AD traits, and the following description of groups are present: Sex, mean age (std), number of samples with RNA-seq, number of samples with proteomics and number of samples with WES.

Supplementary Data 2. Supplementary Data 2 Differential Expression.xlsx

Gene and Protein DE and pathway enrichments for each AD feature as defined in Supplementary Table 2. Pathway enrichments are present only if at least 1 pathway was significantly enriched for corresponding DE genes. Columns are defined in the sheets. The first sheet contains descriptions and hyperlinks to all sheets in the Table.
The gene DE results per trait are in the following sheets: geneDE_Path.Dx, geneDE_CDR, geneDE_PlaqueMean, geneDE_NP.1, geneDE_CERJ, geneDE_bbscore, geneDE_Path.Dx_defvsctl, geneDE_CDR_geneDEfvsctl, geneDE_PlaqueMean_geneDEfvsctl, geneDE_NP.1_geneDEfvsctl and geneDE_CERJ_geneDEfvsctl.
The protein DE results per trait are in the following sheets: protDE_PlaqueMean , protDE_Path.Dx, protDE_NP.1, protDE_CERJ, protDE_CDR, protDE_bbscore, protDE_PlaqueMean_protDEfvsctl, protDE_Path.Dx_protDEfvsctl, protDE_NP.1_protDEfvsctl, protDE_CERJ_protDEfvsctl and protDE_CDR_protDEfvsctl.
The GO pathway enrichments are in the following sheets where the name of the sheet contains the differential expression for a specific AD trait: geneDE_CDR_GO, geneDE_PlaqueMean_GO, geneDE_CERJ_GO, geneDE_Path.Dx_geneDEfvsctl_GO, geneDE_CDR_geneDEfvsctl_GO, geneDE_PlaqueMean_geneDEfvsctl_GO, geneDE_NP.1_geneDEfvsctl_GO and geneDE_CERJ_geneDEfvsctl_GO, protDE_PlaqueMean_GO, protDE_Path.Dx_GO, protDE_NP.1_GO, protDE_CERJ_GO, protDE_CDR_GO, protDE_bbscore_GO, protDE_PlaqueMean_protDEfvsctl_GO, protDE_Path.Dx_protDEfvsctl_GO, protDE_NP.1_protDEfvsctl_GO and protDE_CERJ_protDEfvsctl_GO.
The GSEA pathway enrichments are in the following sheets where the name of the sheet contains the differential expression for a specific AD trait: geneDE_PlaqueMean_GSEA, geneDE_PlaqueMean_geneDEfvsctl_GSEA, protDE_PlaqueMean_GSEA, protDE_CDR_GSEA, protDE_bbscore_GSEA, protDE_PlaqueMean_protDEfvsctl_GSEA, protDE_NP.1_protDEfvsctl_GSEA and protDE_CERJ_protDEfvsctl_GSEA.

Supplementary Data 3. Supplementary Data 3 Networks.xlsx

Network results: Co-expression and Bayesian networks KDA results and RF Z-score ranks for gene, protein and multiscale traits. Columns are defined in the sheets. The first sheet contains descriptions and hyperlinks to all sheets in the Table.

The sheets contain the following results: Gene_coexpression is the gene co-expression network; Gene_Coexpression_GO is the GO annotation for each module of the gene co-expression network; Protein_Coexpression is the protein co-expression network; Protein_Coexpression_GO is the GO annotation for each module of the protein co-expression network; Multiscale_Coexpression is the multiscale co-expression network; Multiscale_Coexp_Breakdown is a table detailing for each module of the multiscale co-expression network the number of genes and protein it contains; Union_DE_genes contains the union of DE genes across all AD traits at FDR<0.05 that is projected onto the gene co-expression network; Union_DE_proteins contains the union of DE proteins across all AD traits at FDR<0.05 that is projected onto the protein co-expression network; signif_modules_gene is a table with the gene co-expression modules enriched for the union of DE genes; signif_modules_protein is a table with the protein co-expression modules enriched for the union of DE proteins; Seeding_gene_list_before_PEXA is the expanded AD DE signature containing all DE genes and all genes in gene co-expression modules enriched for DE genes; Seeding_gene_list_after_PEXA is the seeding gene list from which the BN are constructed, containing all DE genes and all genes in gene co-expression modules enriched for DE genes as well as genes added from protein-protein interaction networks and KEGG pathways; Gene_BN is the gene only BN; Protein_BN is the protein only BN and Multiscale_BN is the multiscale BN. KDA_Gene_BN_ordered is the results of local KDA for all AD traits gene DE lists and gene co-expression modules in the gene BN ordered by their information Z-score rank; KDA_Protein_BN_ordered is the results of local KDA for all AD traits protein DE lists and protein co-expression modules in the protein BN ordered by their information Z-score rank; KDA_Multiscale_BN_ordered is the results of local KDA for all AD traits gene and protein DE lists and gene and protein co-expression modules in the multiscale BN ordered by their information Z-score rank; KDA_Gene_BN_DE_ordered is the results of local KDA for all AD traits gene DE lists in the gene BN ordered by their information Z-score rank; KDA_Protein_BN_DE_ordered is the results of local KDA for all AD traits protein DE lists in the protein BN ordered by their information Z-score rank; KDA_Multiscale_BN_DE_ordered is the results of local KDA for all AD traits gene and protein DE lists in the multiscale BN ordered by their information Z-score rank; Gene_BN_global_KDs is a list of the global KDs for the gene BN; Protein_BN_global_KDs is a list of the global KDs for the protein BN; Multiscale_BN_global_KDs is a list of the global KDs for the multiscale BN. Gene_RF_rank is the ranks from the weighted z-score random forest classifiers for every gene in the gene expression data; Protein_RF_rank is the ranks from the weighted z-score random forest classifiers for every protein in the protein expression data; Multiscale_RF_rank is the ranks from the weighted z-score random forest classifiers for every gene and protein in the gene and protein expression data.

Supplementary Data 4. Supplementary Data 4 QTLs.xlsx

QTL results: eQTL, pQTL and CIT results. Columns are defined in the sheets. The first sheet contains descriptions and hyperlinks to all sheets in the Table.

The sheets contain the following results: eGenes is a table of eGene results with each line being a gene and its most significant eQTL; eProteins is a table of eProtein results with each line being a protein and its most significant pQTL; common_e_p_QTL is a table defining the e- and p-QTL under shared SNP control; eGenes_forNetwork is a table containing the eGenes that were included in the BNs; eProteins_forNetwork is a table containing the eProteins that were included in the BNs; CIT_gene_to_protein is a table with the results of the CIT test for genes and proteins under shared SNP control for the gene parent model; CIT_protein_to_gene is a table with the results of the CIT test for genes and proteins under shared SNP control for the protein parent model (reactive model).

Supplementary Data 5. Supplementary Data 5 Replication.xlsx

Replication results in BM22, BM44, BM36 and ROSMAP. Columns are defined in the sheets. The first sheet contains descriptions and hyperlinks to all sheets in the Table.

The DE results per trait are in the following sheets (only genes with FDR<0.05 are shown): BM22_DE_bbscore, BM22_DE_CDR, BM22_DE_CDR_defvsctl, BM22_DE_CERJ, BM22_DE_CERJ_defvsctl, BM22_DE_NP.1, BM22_DE_NP.1_defvsctl, BM22_DE_PATH.Dx, BM22_DE_PATH_Dx_defvsctl, BM22_DE_PlaqueMean, BM22_DE_PlaqueMean_defvsctl, BM44_DE_bbscore, BM44_DE_CDR, BM44_DE_CDR_defvsctl, BM44_DE_CERJ, BM44_DE_CERJ_defvsctl, BM44_DE_NP.1, BM44_DE_NP.1_defvsctl, BM44_DE_PATH.Dx, BM44_DE_PATH_Dx_defvsctl, BM44_DE_PlaqueMean, BM44_DE_PlaqueMean_defvsctl, BM36_DE_bbscore, BM36_DE_CDR, BM36_DE_CDR_defvsctl, BM36_DE_CERJ, BM36_DE_CERJ_defvsctl, BM36_DE_NP.1, BM36_DE_NP.1_defvsctl, BM36_DE_PATH.Dx, BM36_DE_PATH_Dx_defvsctl, BM36_DE_PlaqueMean, BM36_DE_PlaqueMean_defvsctl, ROSMAP_DE_braaksc, ROSMAP_DE_ceradsc_defvsctl, ROSMAP_DE_ceradsc, ROSMAP_DE_cogdx_defvsctl, ROSMAP_DE_cogdx.

The GO pathway enrichments are in the following sheets where the name of the sheet contains the differential expression for a specific AD trait (only terms with FDR<0.05 are shown): BM22_DE_CERJ_GO, BM22_DE_NP.1_defvsctl_GO, BM22_DE_PlaqueMean_GO, BM22_DE_PlaqueMean_defvsctl_GO, BM36_DE_bbscore_GO, BM36_DE_CDR_GO, BM36_DE_CDR_defvsctl_GO, BM36_DE_CERJ_GO, BM36_DE_CERJ_defvsctl_GO, BM36_DE_NP.1_GO, BM36_DE_NP.1_defvsctl_GO, BM36_DE_PATH.Dx_GO, BM36_DE_PATH_Dx_defvsctl_GO, BM36_DE_PlaqueMean_GO, BM36_DE_PlaqueMean_defvsctl_GO, ROSMAP_DE_braaksc _GO, ROSMAP_DE_ceradsc_defvsctl_GO, ROSMAP_DE_ceradsc _GO, ROSMAP_DE_cogdx_defvsctl_GO, ROSMAP_DE_cogdx _GO. There was no GO pathway enrichments for BM44.

The remaining sheets contain the following results: BM22_Union_DE_genes contains the union of DE genes across all AD traits at FDR<0.05 that is projected onto the gene co-expression network; BM22_Gene_coexpression is the gene co-expression network; BM22_Gene_Coexpression_GO is the GO annotation for each module of the gene co-expression network; BM22_signif_modules_gene is a table with the gene co-expression modules enriched for the union of DE genes; BM22_eGenes is a table of eGene results with each line being a gene and its most significant eQTL; BM22_eGenes_forNetwork is a table containing the eGenes that were included in the BNs; BM22_Seeding_gene_list is the expanded AD DE signature containing all DE genes and all genes in gene co-expression modules enriched for DE genes;

BM22_Gene_BN is the gene only BN; BM22_local_KDA is the results of local KDA for all AD traits gene DE lists and gene co-expression modules in the gene BN; BM22_global_KDA is a list of the global KDs for the gene BN; BM44_Union_DE_genes contains the union of DE genes across all AD traits at FDR<0.05 that is projected onto the gene co-expression network; BM44_topCorGenes is a table of all the union DE genes and the union of their top 10 genes most correlated genes as defined in the methods (Seeding gene list construction); BM44_Gene_coexpression is the gene co-expression network; BM44_Gene_Coexpression_GO is the GO annotation for each module of the gene co-expression network; BM44_signif_modules_gene is a table with the gene co-expression modules enriched for the union of DE genes and their top 10 correlated genes; BM44_eGenes is a table of eGene results with each line being a gene and its most significant eQTL; BM44_ eGenes_forNetwork is a table containing the eGenes that were included in the BNs; BM44_Seeding_gene_list is the expanded AD DE signature containing all DE genes and all genes in gene co-expression modules enriched for DE genes; BM44_Gene_BN is the gene only BN; BM44_local_KDA is the results of local KDA for all AD traits gene DE lists and gene co-expression modules in the gene BN; BM44_global_KDA is a list of the global KDs for the gene BN; BM36_Union_DE_genes contains the union of DE genes across all AD traits at FDR<0.05 that is projected onto the gene co-expression network; BM36_Gene_coexpression is the gene co-expression network; BM36_Gene_Coexpression_GO is the GO annotation for each module of the gene co-expression network; BM36_signif_modules_gene is a table with the gene co-expression modules enriched for the union of DE genes; BM36_eGenes is a table of eGene results with each line being a gene and its most significant eQTL; BM36_eGenes_forNetwork is a table containing the eGenes that were included in the BNs; BM36_Seeding_gene_list is the expanded AD DE signature containing all DE genes and all genes in gene co-expression modules enriched for DE genes; BM36_Gene_BN is the gene only BN; BM36_local_KDA is the results of local KDA for all AD traits gene DE lists and gene co-expression modules in the gene BN; BM36_global_KDA is a list of the global KDs for the gene BN; ROSMAP_Union_DE_genes contains the union of DE genes across all AD traits at FDR<0.05 that is projected onto the gene co-expression network; ROSMAP_Gene_coexpression is the gene co-expression network; ROSMAP_Gene_Coexpression_GO is the GO annotation for each module of the gene co-expression network; ROSMAP_signif_modules_gene is a table with the gene co-expression modules enriched for the union of DE genes; ROSMAP_eGenes is a table of eGene results with each line being a gene and its most significant eQTL; ROSMAP_eGenes_forNetwork is a table containing the eGenes that were included in the BNs; ROSMAP_Seeding_gene_list is the expanded AD DE signature containing all DE genes and all genes in gene co-expression modules enriched for DE genes; ROSMAP_Gene_BN is the gene only BN; ROSMAP_local_KDA is the results of local KDA for all AD traits gene DE lists and gene co-expression modules in the gene BN; ROSMAP_global_KDA is a list of the global KDs for the gene BN.

Supplementary Data 6. Supplementary Data 6 Mouse Differential Expression.xlsx

Results of the mouse DE for both molecular validation models (genes with FDR<0.05 are indicated in bold). Columns are defined in the sheets. The first sheet contains descriptions and hyperlinks to all sheets in the Table.

The sheets contain the following results: FADAAVGFP.FADAAVVGF is the results if the DE between 5xFAD,AAV-VGF and 5xFAD,AAV-GFP (genes with FDR<0.05 are indicated in

bold); FADVGFhom.FADVGFwt is the results of the DE between 5xFAD,$VGF^{\Delta/\Delta}$ and 5xFAD with normal levels of VGF (genes with FDR<0.05 are indicated in bold).
